# Delaying *Candidatus* Liberibacter asiaticus infection of citrus trees through use of individual protective covers and systemic delivery of oxytetracycline

**DOI:** 10.3389/fpls.2025.1671217

**Published:** 2025-10-28

**Authors:** Caroline Tardivo, Brittney Monus, Gabriel Pugina, Sarah L. Strauss, Fernando Alferez, Ute Albrecht

**Affiliations:** ^1^ Horticultural Sciences Department, University of Florida/IFAS, Southwest Florida Research and Education Center, Immokalee, FL, United States; ^2^ Department of Soil, Water, and Ecosystem Sciences, University of Florida/IFAS, Southwest Florida Research and Education Center, Immokalee, FL, United States

**Keywords:** citrus greening, vector control, bacterial disease, antibiotics, endotherapy, endorhizosphere, bacterial community

## Abstract

Huanglongbing (HLB), or citrus greening, remains one of the most destructive diseases affecting citrus production globally. Associated with the phloem-limited bacterium *Candidatus* Liberibacter asiaticus (*C*Las) and vectored by *Diaphorina citri*, HLB leads to canopy decline, fibrous root loss, and reductions in fruit yield and quality. Recently, the systemic delivery of oxytetracycline (OTC) via trunk injection was approved in Florida as a targeted therapy to reduce *C*Las titers and improve tree health. In parallel, Individual Protective Covers (IPCs) have been adopted to delay *C*Las infection in newly planted citrus trees by vector exclusion. This study investigates the combined use of IPCs and trunk injection of OTC for post-IPC therapy. ‘Valencia’ sweet orange trees grafted on US-812 and US-942 rootstocks were planted in December 2020 under HLB-endemic conditions in southwest Florida. IPCs were installed at planting and removed after 18 months. The first OTC injection was performed in May 2023, 10 months after IPC removal. A second injection was performed in May 2024. A 2 × 2 × 2 factorial experimental design evaluated the effects of infection history (early-infected and late-infected), rootstock cultivar (US-812 and US-942), and injection treatment (OTC-injected and non-injected) on tree responses over two consecutive production seasons. In year 1, infection history significantly influenced tree size, fruit yield, total soluble solids (TSS), TSS/titratable acidity ratio, and peel color. Late-infected trees outperformed early-infected trees, regardless of injection treatment and rootstock cultivar. In year 2, OTC-injected trees exhibited significantly higher yields, improved juice quality, and enhanced canopy health regardless of infection history and rootstock cultivar. Fibrous root microbiome analyses based on 16S rRNA sequencing revealed no significant effects of OTC injection on bacterial alpha or beta diversity, with stable community structure observed across treatments and time points. This suggests that targeted vascular delivery of OTC may not cause any major disruption to the root endorhizosphere microbiome. Together, the results from this study demonstrate the efficacy of integrating preventative (use of IPCs) and therapeutic (OTC vascular delivery) strategies for sustainable HLB management while preserving microbial integrity and offering a model for citrus production in parts of the world where HLB is prevalent.

## 1 Introduction

Huanglongbing (HLB), also called citrus greening, has devastated citrus industries around the world, especially in Florida, where it was discovered in 2005 and declared endemic by 2013 (Gottwald et al., 2007; Graham et al., 2020 and 2024). The disease is associated with *Candidatus* Liberibacter asiaticus (*C*Las), a phloem-restricted bacterium transmitted by the Asian citrus psyllid (*Diaphorina citri*) (Halbert and Manjunath, 2004). Infection results in drastic reductions in tree health, fruit yield, and fruit quality (Bové, 2006; Gottwald et al., 2007; Da Graça et al., 2015). The primary strategy to manage HLB by controlling the psyllid vector is not only costly but has become ineffective, as psyllids have developed resistance to the available insecticides (Tiwari et al., 2011; Stansly et al., 2014). A promising strategy for preventing the spread of HLB to young citrus trees is the use of Individual Protective Covers (IPCs). IPCs are composed of polyethylene mesh bags with a mesh size smaller than the size of a psyllid. These covers prevent the vector from accessing the new flushes and consequently, from transmitting *C*Las, thus delaying the onset of infection during the critical early years of tree establishment (Alferez et al., 2021). Recent studies documented that trees protected with IPCs remain *C*Las-free and healthy under HLB-endemic conditions and produce more and better-quality fruits than non-protected trees (Gaire et al., 2022 and 2023). One of the early consequences of *C*Las infection is the loss of fibrous roots (Johnson et al., 2014; Tardivo et al., 2024), which, especially in young trees, contributes to rapid whole-tree decline. In our previous research, infected trees had a 37% reduction in total root biomass, with fibrous root loss reaching 49% and canopy biomass declining by 20% compared to non-infected trees (Tardivo et al., 2024).

Despite their benefits, IPCs are not a permanent solution as they must be removed once the canopy has become too large, resulting in *C*Las infection within a few months under Florida growing conditions (Gaire et al., 2024). Given the rapid infection and the associated fibrous root loss, effective post-IPC interventions are needed to sustain tree health and productivity. One promising approach to retain tree health is the systemic delivery of oxytetracycline (OTC) by trunk injection, which has recently emerged as a targeted strategy to suppress *C*Las in infected trees (Albrecht et al., 2025a). This technique delivers the antibiotic directly into the tree’s vascular system, reducing bacterial titers and improving canopy density, fruit and juice quality, and yield (Hu and Wang, 2016; Archer et al., 2022a; 2023). Unlike foliar sprays or soil drenches, trunk injection minimizes environmental contamination and off-target impacts (Wise et al., 2014; Archer et al., 2022b), making it a promising option for HLB management in commercial citrus orchards. The delivery of OTC by trunk injection was approved for commercial use in Florida citrus in 2022 and has been widely adopted since then (Giles, 2025). Trunk injection or infusion of antibiotics and other materials was investigated already in the 1970s in South Africa and other countries to treat HLB-affected citrus trees (Schwarz et al., 1974; Chiu et al., 1978; Van Vuuren, 1977) but was not pursued on a large scale.

While OTC injection has demonstrated efficacy in reducing *C*Las titers and improving tree performance, its broader impacts on the root-associated microbiome remain largely unexplored. Given that fibrous root loss is a major consequence of *C*Las infection, understanding how OTC influences the endorhizosphere is important for evaluating its long-term sustainability as a post-IPC management strategy. Recent studies have shown that OTC injection significantly alters the active microbial communities in both the bark and rhizosphere, temporarily reducing bacterial diversity and abundance while specifically enriching taxa potentially related to improved tree performance (Castellano-Hinojosa et al., 2024). However, whether these microbial shifts extend to the endorhizosphere remains unclear. Understanding these interactions is important for assessing the ecological implications of OTC treatments and addressing concerns about the environmental fate of systemic bactericides.

This study expands on our earlier research with the objective of evaluating an integrated approach to managing HLB in newly established citrus orchards by transitioning from the use of IPCs to the systemic delivery of OTC by trunk injection. We hypothesize that while IPCs delay infection of newly planted citrus trees, OTC injections will reduce disease progression as the trees enter maturity. At the same time, this study investigates the fibrous root microbiome dynamics of citrus trees under this integrated management strategy to assess any potential implications associated with the vascular delivery of OTC. We hypothesize that while effective against *C*Las, OTC injections will have no lasting impact on the overall composition and diversity of the fibrous root bacterial community.

## 2 Materials and methods

### 2.1 Plant material

**Figure 1:**
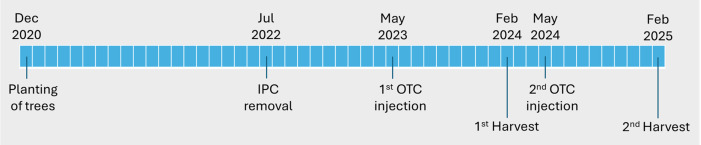
Timeline of experiment.

The trees used in this study were ‘Valencia’ sweet orange (*Citrus sinensis*) grafted on US-812 (‘Sunki’ mandarin [*C. reticulata*] × ‘Benecke’ trifoliate orange [*Poncirus trifoliata*]) or US-942 (‘Sunki’ mandarin × ‘Flying Dragon’ [*P. trifoliata*]) rootstock. Trees were produced in a controlled, HLB-free greenhouse at the University of Florida/IFAS Southwest Florida Research and Education Center (SWFREC) in Immokalee, FL, and transplanted to a field site (26.463663 N, 81.443892 W) at the SWFREC research farm in December 2020. Trees were planted in two rows on a raised bed at a spacing of 6.7 m (22 ft) between rows and 2.44 m (8 ft) within rows. Individual protective covers (IPCs) were used to create non-infected and infected trees as described in Tardivo et al. (2024). Briefly, upon planting, half of the trees were covered with IPCs to protect them from psyllids and *C*Las infection. The other half of the trees were covered with “open IPCs” (IPCs with holes cut into them) that enabled psyllids to access the trees, thereby creating (early-) infected trees. IPCs were removed from the trees in July 2022, 18 months after planting. This resulted in the previously protected, non-infected trees becoming (late-) infected in the months following IPC removal. Thus, two sets of trees with different injection histories (early-infected and late-infected) were created before performing the first OTC injection 10 months after IPC removal (see paragraphs 2.2 and 2.3). The experimental timeline is summarized in **Figure 1**.

Trees were fertilized at a rate of 0.5 pounds (0.23 kg) per tree using conventional granular fertilizer (8N–4P–8K; Diamond R, Fort Pierce, FL, USA) every 6 months and slow-release fertilizer (12N–8P–6K; Diamond R) once per year. Irrigation was performed by under-tree microjets. Insecticides were applied as needed with no psyllid control during the first 18 months (Tardivo et al., 2024), followed by annual dormant and early season foliar applications of broad-spectrum insecticides (Qureshi and Stansly, 2010).

### 2.2 Experimental design

The experiment was a blocked 2 × 2 × 2 factorial design with five single-tree replications. The three factors were: 1) infection history, 2) rootstock cultivar, and 3) injection treatment. The first factor (infection history) had two levels with trees either classified as early-infected (exposed to psyllids and *C*Las since planting), or as late-infected (protected from psyllids and *C*Las for 18 months). The second factor (rootstock cultivar) had two levels with trees grafted on either US-812 or US-942. The third factor (injection treatment) also had two levels, with trees either injected with oxytetracycline (OTC-injected) or not (non-injected). The experimental treatments are summarized in **Table 1**.

**Table 1:** Summary of experimental treatments.

Infection history	Rootstock cultivar	Injection treatment	Number of replications
Early-infected	US-812	OTC-injected	5
		Non-injected	5
	US-942	OTC-injected	5
		Non-injected	5
Late-infected	US-812	OTC-injected	5
		Non-injected	5
	US-942	OTC-injected	5
		Non-injected	5

### 2.3 Oxytetracycline injection

Leaf bacterial titers of all trees were assessed prior to injection (5 and 9 months after IPC removal) as described in paragraph 2.4. Trees were injected with OTC once annually for two consecutive years, starting in May 2023, 10 months after IPC removal (**Figure 1**). Injections were administered into the rootstock trunk, approximately 5 cm below the graft union, using one Chemjet^®^ Tree Injector (Logical Result LLC, Interlochen, MI) per tree. A commercial formulation of oxytetracycline (ReMedium TI^®^, TJ BioTech LLC, Buffalo, SD, USA; 95.0% oxytetracycline hydrochloride) was dissolved in acidified, de-ionized water according to the manufacturer’s instructions. In May 2023, each tree received 0.275 g OTC (active ingredient) dissolved in a volume of 20 ml (13,750 ppm). The dosage was determined based on the average rootstock trunk diameter (6.0 cm) at the time of injection and previous experience. Each tree received the same dose, regardless of its size. Injections were performed by drilling a hole to a depth of 15 mm using a 4.3 mm (11/64 inch) brad point drill bit at an angle of 20–30 degrees. The injectors were inserted, activated, and removed once trees had absorbed the entire 20 mL of dissolved OTC, which took approximately 10–60 minutes. All injections were carried out on a sunny day, between 9 am and 10 am. Trees were injected for a second time in May 2024 following the same procedure. Due to the increased tree size, the dose was increased to 0.55 g of OTC (active ingredient) per tree, and two Chemjet injectors were used to deliver 0.275 g of OTC each on two opposite sides of each tree to ensure adequate distribution and prevent sectoring (Albrecht et al., 2025a). No phytotoxic effects were observed in the canopy in either year following the OTC injections.

### 2.4 *Candidatus* Liberibacter asiaticus detection

Leaves were collected five and nine months after IPC removal (December 2022 and April 2023, respectively). Following OTC injections, leaves and fibrous roots were collected at 3, 30, 60, 90, 180, 270, and 360 days each after the first and second OTC injection (DAI-1 and DAI-2, respectively): 3 DAI-1 (May 2023), 30 DAI-1 (June 2023), 60 DAI-1 (July 2023), 90 DAI-1 (August 2023), 180 DAI-1 (November 2023), 270 DAI-1 (February 2024), 360 DAI-1 (May 2024), 3 DAI-2 (May 2024), 30 DAI-2 (June 2024), 60 DAI-2 (July 2024), 90 DAI-2 (August 2024), 180 DAI-2 (November 2024), 270 DAI-2 (February 2025), and 360 DAI-2 (May 2025). Four leaves per tree were randomly collected from different areas in the canopy and pooled. Leaves were collected from the most recent mature flush and stored at -20 °C until analysis. Fibrous roots (≤ 1.5 mm in diameter) were collected from different areas of the soil underneath the canopy of each tree, washed with water, blotted dry, and stored at -20°C until analysis. Tissues were pulverized with liquid nitrogen using a mortar and pestle. DNA was extracted from leaves and roots using the Plant DNeasy^®^ Pro-Kit (Qiagen) according to the manufacturer’s instructions. Real-time polymerase chain reaction (PCR) assays were performed to detect *C*Las in the leaves and roots using primers HLBas, HLBr, and probe HLBp developed by Li et al. (2006). Primers COXf, COXr, and probe COXp (Li et al., 2006) were used for internal control and normalization. Amplifications were performed over 40 cycles using the iTaq™ Universal Probes Supermix (Bio-Rad, Hercules, CA) and a QuantStudio 3 real-time PCR system (Applied Biosystems, Foster City, CA) according to the manufacturer’s instructions. For samples for which no signal was detected after 40 amplification cycles, a Ct-value of 41 was used for statistical analysis purposes.

### 2.5 Oxytetracycline residue analysis

Oxytetracycline residues in leaves and fibrous roots were analyzed after the first OTC injection in 2023 to understand OTC distribution and movement. Leaves and fibrous roots were collected simultaneously with the *C*Las detection collections at 3, 30, 60, 90, and 180 days after the first injection (DAI-1) and immediately flash-frozen in liquid nitrogen. Leaf and root tissues were pulverized in liquid nitrogen and stored at -80 °C until analysis. OTC detection followed the protocol established by Hijaz et al. (2021) with some modifications. One hundred milligrams of pulverized tissue were added to 1 ml OTC extraction solution (2.2% trichloroacetic acid in 1M HCl). Samples were shaken for 30 minutes with a TissueLyser II (Qiagen, Valencia, California, USA) at 1,400 rpm, and centrifuged for 15 minutes at 1,400 rcf, at 4 °C. Oasis PriME hydrophilic-lipophilic-balanced cartridges (Waters, Milford, MA) were equilibrated with 6 mL of 100% methanol, followed by 6 mL of ultrapure (Milli-Q) water. Two hundred and fifty microliters of the tissue extraction supernatant were transferred into the cartridge, washed with 2 mL of 20% methanol, and eluted with 1 mL of 60% methanol. Eluted tissue extracts were stored in 2 mL amber plastic tubes before fluorescence analysis. Twenty micrograms of the cleaned tissue extracts were added to each well of a 96-well black polystyrene microplate (Corning Inc., Corning, NY). One hundred and eighty microliters of a reaction mix containing 60 µl Tris buffer (0.1 M, pH 8.5), 8 µl citrate (0.0006 M), 3 µl Europium were added to each well and mixed by pipetting. The microplate was incubated in the dark for 30 minutes. Fluorescence was measured following the method described in Hijaz et al. (2021) using a multimode reader (BioTek Synergy HTX, Santa Clara, CA) with the first wavelength (excitation) set at 360 ± 40 nm, the second wavelength (emission) set at 620 ± 15 nm, and the gain wavelength set at 80 nm.

### 2.6 Tree size and canopy health

Tree biometric measurements, visual ratings of canopy health, and leaf size measurements were conducted in November 2024. Tree height was measured from the base of the trunk to the top of the canopy using a measuring tape. The canopy diameter was measured in two perpendicular directions, east-west and north-south, using a measuring tape, averaged, and the canopy volume was determined by multiplying tree height and canopy width, and dividing the product by 4 (Wutscher and Hill, 1995). Scion and rootstock trunk diameters were measured 5 cm above and below the graft union.

Tree health assessments included visual ratings of canopy density and foliar symptoms of HLB, leaf size-, and leaf color (chlorophyll content) measurements. Canopy density was rated on a scale of 1 to 5, where 1 = very sparse and 5 = very dense. HLB severity was rated on a scale of 1 to 5 where 1 = 0% of branches with HLB symptoms, 2 = 0-25% of branches with HLB symptoms, 3 = 25-50% of branches with HLB symptoms, 4 = 50-75% of branches with HLB symptoms, and 5 = > 75% of branches with HLB symptoms. HLB disease symptoms were defined as irregular blotchy mottling of the leaves typical for HLB (Gottwald et al., 2007). The average leaf size was determined by scanning 10 randomly selected leaves per tree at 400 dpi on a flatbed scanner (Epson Perfection v850 Pro; Epson America, Los Alamitos, CA) and calculating the leaf area using ImageJ 1.52p software (Rasband, NIH, USA). Leaf chlorophyll content was measured on the same leaves using a SPAD 502 Plus Chlorophyll Meter (Spectrum Technologies, Aurora, IL).

### 2.7 Fruit yield, pre-harvest fruit drop, fruit quality, and juice quality

Fruit yield and fruit/juice quality were determined at harvest at the end of February in each year (26 February 2024, and 14 February 2025). Fruits were harvested from each tree and weighed, and yield was expressed in kg of fruits per tree. Pre-harvest fruit drop was determined at harvest by counting fruits on the ground and expressed as a percentage relative to the sum of dropped plus retained fruits. A random set of 10 fruits from each tree was used to measure the fruit size, fruit weight, peel color, and juice quality. Fewer fruits were used when trees did not produce enough fruits. Fruit size was determined using a Mitutoyo Digital Caliper (Mitutoyo America Corporation, Aurora, IL) by measuring the width and height of each fruit, and the volume was calculated using the formula for the volume of a sphere and expressed in cm³.

Fruits were juiced using an automatic feed juicer (Juicernet JM-30, Jupiter, FL). Juice samples were used to measure total soluble sugars (TSS) using a digital refractometer (Hanna Instruments, Smithfield, RI) and titratable acidity (TA) by titrating sodium hydroxide to a phenolphthalein endpoint, and the TSS/TA ratio was determined. Peel color was determined using a CR-400 chroma meter (Konica Minolta, Ramsey, NJ) and presented as the a*/b* color ratio based on the CIE L*a*b* color system. Negative a*/b* ratio values indicate a deeper green color, and positive values indicate a deeper red (orange) color (Bai et al., 2009).

### 2.8 Leaf nutrient content

Leaf nutrients were analyzed in July 2023 and July 2024. Twenty mature leaves from the most recent spring flush were collected randomly from each tree. Analysis of macro- (N, P, K, Ca, Mg, S) and micronutrients (B, Zn, Mn, Fe, Cu) was conducted by Waters Agricultural Laboratories, Inc. (Camilla, GA). Total nitrogen was determined using the combustion method described by Sweeney (1989). The leaves were then digested with nitric acid and hydrogen peroxide to determine the other macro- and micronutrients using inductively coupled argon plasma atomic emission spectroscopy (Havlin and Soltanpour, 1980; Huang and Schulte, 1985).

### 2.9 Root endorhizosphere bacterial community

Only trees on US-942 rootstock were included for the root endorhizosphere bacterial community analysis. US-942 was the most popular rootstock in Florida at the initiation of this study (FDACS, 2024).

#### 2.9.1 Sample collection and RNA extractions

Fibrous root samples were collected as described for *C*Las analysis. Samples were collected at 30, 90, and 270 days after the first injection (DAI-1), i.e., in June 2023, August 2023, and February 2024, respectively. Fibrous roots were immediately frozen in liquid nitrogen and stored at −80 °C until analysis. Root tissue was ground in liquid nitrogen using a mortar and pestle. Total RNA was extracted from 100 mg of ground tissue using the RNA Powerkit^®^ Total RNA Isolation kit (Qiagen). RNA concentrations were quantified using the Qubit™ RNA High Sensitivity assay kit (Thermo Scientific, Waltham, MA, USA). Co-extracted DNA was removed using DNase I (RNase free, Qiagen), following the manufacturer’s directions. Complementary DNA (cDNA) synthesis was performed with the SuperScript IV First-Strand Synthesis System with ezDNase following the manufacturer’s instructions. cDNA concentrations were quantified using the Qubit™ DNA High Sensitivity assay kit and stored at −80 °C for subsequent analyses.

#### 2.9.2 Library preparation and sequencing

The extracted cDNA was submitted to the Genomics and Microbiome Core Facility at Rush University (Chicago, IL) for sequencing. The V4 region of the bacterial 16S rRNA gene was amplified using the 515Fa (GTGYCAGCMGCCGCGGTAA) and 926R (CCGYCAATTYMTTTRAGTTT) primers following the Earth Microbiome Project protocol (Apprill et al., 2015). The sequencing was conducted on an Illumina NovaSeq platform to generate paired-end reads. After sequencing, raw reads were processed using Quantitative Insights Into Microbial Ecology (QIIME) version 2 (Bolyen et al., 2019). Reads were assembled and dereplicated using DADA2 (Callahan et al., 2019), with paired-end settings generating amplicon sequencing variants (ASVs). ASVs were assigned taxonomically using the SILVA 138 database and a naïve Bayes classifier within QIIME2 (Bokulich et al., 2018; Quast et al., 2012). Non-bacterial sequences (mitochondria and chloroplasts) were removed, and samples with fewer than 1,000 reads were excluded from the analysis.

### 2.10 Data analysis

#### 2.10.1 Tree biometric analysis

A linear mixed model in R programming (Version 4.3, R Core Team, 2024) was used with infection history, rootstock cultivar, and injection treatment as fixed factors and block as a random factor. The Shapiro-Wilk tests and Q-Q plots were employed to assess assumptions of normality and homogeneity of distribution of the data. When assumptions were not met, data were log-transformed. *C*Las titers (Ct-values) were analyzed for each sampling time and each tissue (leaves and roots). Where effects were significant (P < 0.05), a *post-hoc* comparison of means was conducted using Tukey’s honestly significant test (emmeans). Visual ratings of canopy health were analyzed non-parametrically using aligned ranks transformation ANOVA (ART). Principal component analysis (PCA) was performed on standardized data to reduce the influence of different measurement scales among variables (Sneath and Sokal, 1973). Contribution analysis was included to assess the relative importance of individual variables. Cluster analyses were conducted using the NbClust package in R version 4.3 (Charrad et al., 2014), while PCA was performed using the FactoMineR package (Lê et al., 2008). Variables included in the PCA were selected based on their statistical significance. Only data from year 2 (after the second OTC injection) were included in the PCA.

#### 2.10.2 Endorhizosphere bacterial community

Alpha diversity metrics, including observed richness, Shannon diversity index, and Simpson diversity index, were calculated using the phyloseq package (McMurdie and Holmes, 2013). Differences in alpha diversity metrics between groups (late-infected vs. early-infected and OTC-injected vs. non-injected) were assessed using non-parametric Wilcoxon rank-sum tests with a significance threshold of p < 0.05. Beta diversity was assessed using Bray-Curtis dissimilarity matrices. Principal Coordinates Analysis (PCoA) was conducted to visualize bacterial community differences, and the first two principal components were reported. Group differences in beta diversity were tested using PERMANOVA in the vegan package (Oksanen et al., 2020), with 999 permutations to estimate the variance explained (R²) and statistical significance (p < 0.05). Pairwise PERMANOVA comparisons were conducted as necessary to evaluate specific group interactions.

## 3 Results

### 3.1 *Candidatus* Liberibacter asiaticus detection

*C*Las titers are expressed as cycle threshold (Ct) values with low values representing high bacterial titers and high values representing low bacterial titers.

#### 3.1.1 Leaves

Five months after IPC removal (December 2022), 35% of late-infected trees had Ct-values ≥ 36, suggesting they were not yet *C*Las-infected. All other trees had Ct-values ≤ 36, indicating they were infected. The average Ct-value of late-infected trees was significantly higher (36.2), and therefore bacterial titers lower, compared to early-infected trees (20.1) (**Table 2**). Nine months after IPC removal (April 2023), just prior to the first OTC injection, Ct-values ranged from 22.5 to 24.1, indicating that all trees were infected, with no significant difference between late- and early-infected trees. There were no significant rootstock effects or block effects, nor were there any significant interactions at either time.

**Table 2:** Ct-values of leaves from ‘Valencia’ trees on different rootstocks and with different infection histories in December 2022 and April 2023 before the first OTC injection.

Factor	December 2022	April 2023
Infection history		
Late-infected	36.2 a	24.1
Early-infected	20.1 b	22.5
*p-value*	*<0.0001*	*0.9030*
Rootstock cultivar		
US-812	28.5	23.1
US-942	27.8	23.5
*p-value*	*0.6118*	*0.6644*
Infection history × Rootstock cultivar		
*p-value*	*0.6052*	*0.8080*
Block		
*p-value*	*0.3082*	*1.0000*

Different letters within columns indicate significant differences according to Tukey’s honestly significant difference test. Letters are not shown when P > 0.05.

**Figure 2:**
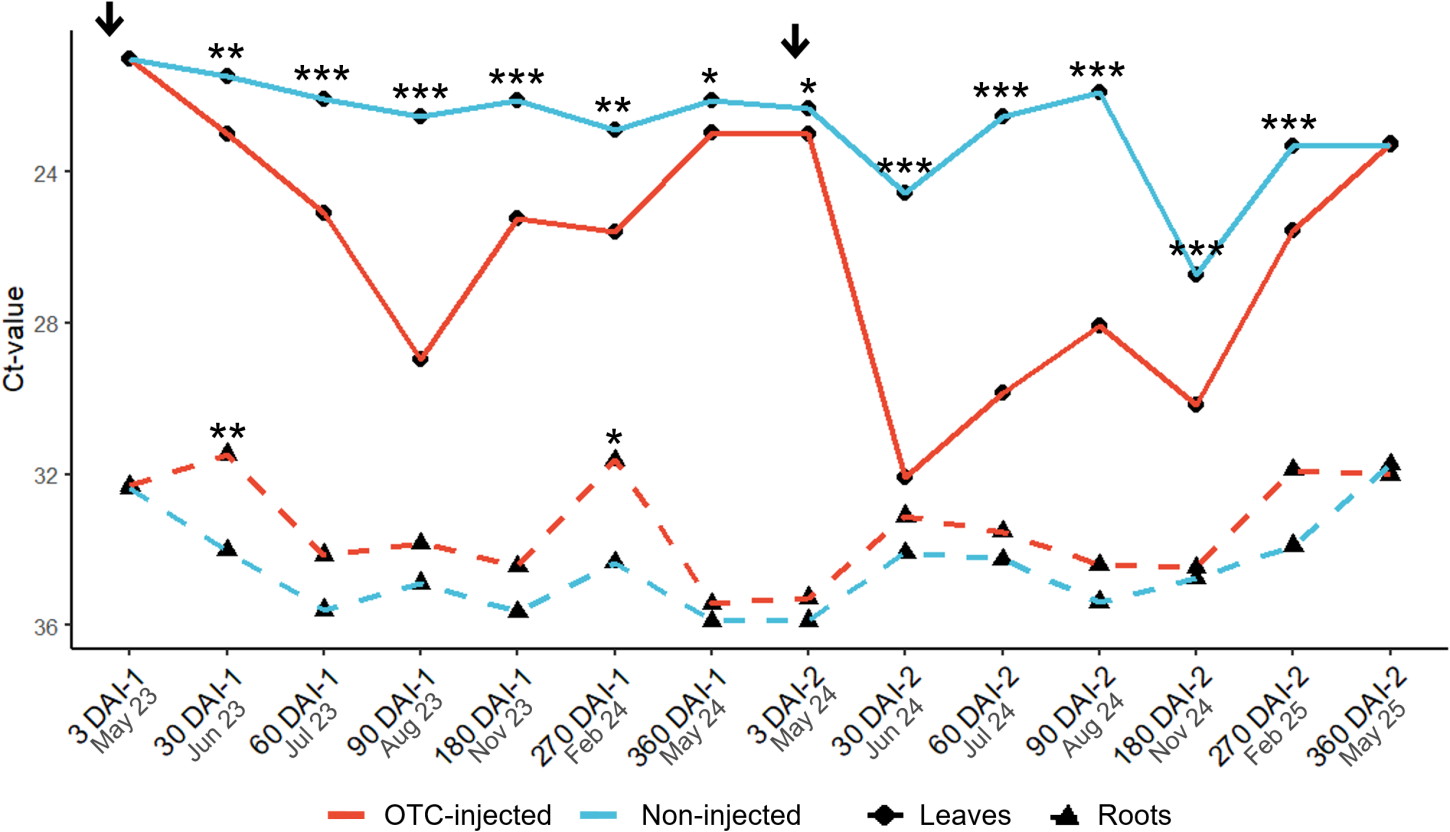
Graphical depiction of leaf and root Ct-values of OTC-injected and non-injected ‘Valencia’ trees. A low Ct-value indicates high bacterial titers and vice versa. Asterisks indicate statistically significant differences (*, P < 0.05; **, P < 0.01; ***, P <0.001) between OTC-injected and non-injected trees for each tissue. Arrows indicate the time of injections. For statistical details, see **Tables 2** and **3**. DAI-1 and DAI-2 are days after the first and second injection, respectively.

There was a significant effect of the infection history on the leaf Ct-value after the first OTC-injection (**Table 3**; **Figure 2**). At 3 DAI-1, late-infected trees had a significantly higher leaf Ct-value (21.7) and, therefore, lower leaf bacterial titers than early-infected trees (20.3). There were no significant differences between late- and early-infected trees at any other time except 360 DAI-1, although the mean separation was not significant. No significant differences in Ct-value were observed at any time between the two rootstock cultivars, but there was a significant effect of injection treatment. OTC-injected trees had higher leaf Ct-values (23.0-28.9) and therefore, lower leaf *C*Las titers than non-injected trees (21.5-22.9) from 30 DAI-1 to 360 DAI-1, with the largest differences found at 90 DAI-1 (**Figure 2**). A significant interaction between infection history and injection treatment was observed at 30 DAI, when early-infected, OTC-injected trees exhibited a higher leaf Ct-value (23.8) than early-infected, non-injected trees (20.2), and at 360 DAI, when early-infected, OTC-injected trees exhibited higher Ct-value (23.8) than early-infected, non-injected trees (20.2) (**Supplementary Table S1**). The three-way interaction between infection history, rootstock cultivar, and injection treatment was significant at 360 DAI, but there was no significant mean separation (**Supplementary Table S1**). There were no significant block effects.

**Table 3:** Ct-values of leaves from ‘Valencia’ trees on different rootstocks with different infection histories and injection treatments 3 to 360 days after the first OTC injection (DAI-1).

Factor	Days after infection						
	3 (May 2023)	30 (Jun 2023)	60 (Jul 2023)	90 (Aug 2023)	180 (Nov 2023)	270 (Feb 2024)	360 (May 2024)
Infection history							
Late-infected	21.7 a	22.5	23.9	26.2	23.8	24.4	22.1 a
Early-infected	20.3 b	22.0	23.3	25.3	23.6	24.1	23.0 a
*p-value*	*0.0157*	*0.3343*	*0.8587*	*0.2384*	*0.7751*	*0.7902*	*0.0312*
Rootstock cultivar							
US-812	21.1	21.9	24.3	26.5	23.7	24.3	22.3
US-942	20.9	22.6	22.9	25.0	23.6	24.2	22.8
*p-value*	*0.8999*	*0.2044*	*0.0714*	*0.0631*	*0.8966*	*0.9576*	*0.1886*
Injection treatment							
OTC-injected	21.0	23.0 a	25.1 a	28.9 a	25.2 a	25.6 a	23.0 a
Non-injected	21.0	21.5 b	22.1 b	22.5 b	22.1 b	22.9 b	22.1 b
*p-value*	*0.5645*	*0.0075*	*<0.0001*	*<0.0001*	*0.0003*	*0.0075*	*0.0243*
Infection history × Rootstock cultivar							
*p-value*	*0.1494*	*0.3935*	*0.5407*	*0.2051*	*0.1537*	*0.0894*	*0.0821*
Infection history × Injection treatment							
*p-value*	*0.6512*	*0.0004*	*0.3442*	*0.3594*	*0.2224*	*0.0981*	*0.0446*
Rootstock cultivar × Injection treatment							
*p-value*	*0.9800*	*0.1386*	*0.1407*	*0.0314*	*0.5435*	*0.5357*	*0.0559*
Infection history × Rootstock cultivar × Injection treatment							
*p-value*	*0.4505*	*0.0887*	*0.6444*	*0.0605*	*0.8345*	*0.2007*	*0.0404*
Block							
*p-value*	*0.6679*	*0.7677*	*1.0000*	*1.0000*	*0.6521*	*1.0000*	*1.0000*

Different letters within columns indicate significant differences according to Tukey’s honestly significant difference test. Letters are not shown when P > 0.05.

There was no significant effect of infection history and rootstock cultivar on the leaf Ct-values at any point after the second OTC injection (**Table 4**; **Figure 2**). In contrast, there was a significant injection treatment effect. Leaf Ct-values of OTC-injected trees were higher than those of non-injected trees at all times, except 360 DAI-2. From 3 DAI-2 to 270 DAI-2 Ct-values of OTC-injected trees ranged from 23.0 to 32.0 while those of non-injected trees ranged from 21.8 to 26.8, indicating significant reductions in bacterial titers in response to OTC injection. The largest differences were found between 30 and 90 DAI-2. There were no significant interactions except at 90 DAI-2, when OTC-injected trees on US-812 had higher Ct-values (30.3) than those on US-942 (25.7); at 270 DAI-2, between infection history and injection treatment, when early-infected, OTC-injected trees had higher Ct-values (26.9) than the other trees; and at 360 DAI-2, between infection history and injection treatment with no significant mean separation; and between infection history, rootstock cultivar, and injection treatment, with no significant mean separation (data not shown). No significant block effects were found.

**Table 4:** Ct-values of leaves from ‘Valencia’ trees on different rootstocks with different infection histories and injection treatments 3 to 360 days after the second OTC injection (DAI-2).

Factor	Days after infection						
	3 (May 2024)	30 (Jun 2024)	60 (Jul 2024)	90 (Aug 2024)	180 (Nov 2024)	270 (Feb 2025)	360 (May 2025)
Infection history							
Late-infected	22.4	28.5	25.4	25.5	28.7	23.9	23.2
Early-infected	23.0	28.0	27.0	24.2	28.2	25.0	23.4
*p-value*	*0.1467*	*0.7770*	*0.1702*	*0.2692*	*0.6961*	*0.0625*	*0.7036*
Rootstock cultivar							
US-812	22.7	28.8	26.5	26.0	28.5	24.3	23.7
US-942	22.7	27.7	25.9	23.7	28.4	24.5	22.9
*p-value*	*0.9431*	*0.5061*	*0.6511*	*0.0595*	*0.8725*	*0.7240*	*0.1312*
Injection treatment							
OTC-injected	23.0 a	32.0 a	29.8 a	28.0 a	30.2 a	25.5 a	23.3
Non-injected	22.3 b	24.5 b	22.5 b	21.8 b	26.8 b	23.3 b	23.3
*p-value*	*0.0408*	*<0.0001*	*<0.0001*	*<0.0001*	*0.0005*	*0.0008*	*0.9516*
Infection history × Rootstock cultivar							
*p-value*	*0.8548*	*0.6500*	*0.6985*	*0.4336*	*0.3121*	*0.0982*	*0.8137*
Infection history × Injection treatment							
*p-value*	*0.9640*	*0.9316*	*0.1406*	*0.8224*	*0.0504*	*0.0203*	*0.1171*
Rootstock cultivar × Injection treatment							
*p-value*	*0.5941*	*0.4513*	*0.4203*	*0.0439*	*0.4442*	*0.8544*	*0.0223*
Infection history × Rootstock cultivar × Injection treatment							
*p-value*	*0.4187*	*0.3744*	*0.9464*	*0.7526*	*0.8402*	*0.4624*	*0.1105*
Block							
*p-value*	*0.5841*	*1.0000*	*0.0806*	*0.3364*	*1.0000*	*1.0000*	*1.0000*

Different letters within columns indicate significant differences according to Tukey’s honestly significant difference test. Letters are not shown when P > 0.05.

#### 3.1.2 Roots

Early-infected trees exhibited a significantly higher fibrous root Ct-value at 3 DAI-1 (34.1) and at 60 DAI-1 (36.2) than late-infected trees (30.6 and 33.6, respectively) (**Supplementary Table S2**; **Figure 2**). There was a significant rootstock effect at 180 DAI-1 when US-812 roots had a significantly higher Ct-value (36.3) than US-942 roots (33.8). Injection treatment was significant at 30 DAI-1 and at 270 DAI-1 when OTC-injected trees had lower root Ct-values (31.6 and 31.6, respectively) than non-injected trees (34.01 and 34.3, respectively). There was a significant interaction between infection history and rootstock cultivar at 90 DAI-1, when US-812 roots from early-infected trees exhibited a higher Ct-value (36.9) than US-812 roots from late-infected trees (31.5) (data not shown). There were no significant effects of infection history, rootstock cultivar, or injection treatment on the fibrous root Ct-value at any time after the second injection (**Supplementary Table S3**; **Figure 2**). There were also no significant block effects.

### 3.2 Oxytetracycline residue analysis

Leaf OTC residues were highest at 3 DAI-1 (1.63-2.82 μg/g) and lowest at 180 DAI-1 (0.28-0.35 μg/g) (**Table 5**). Infection history and rootstock cultivar did not significantly affect leaf OTC residues, except at 90 DAI, when trees on US-812 rootstock had higher leaf OTC residues (0.72 μg/g) than those on US-942 (0.43 μg/g). There was no block effect, nor were there any significant interactions. Fibrous root residues were below 0.06 μg/g, and there was no significant effect of any of the factors or their interaction (**Table 5**). No residues were detected between 90 and 180 DAI-1 (data not shown).

**Table 5:** Leaf and fibrous root OTC residues (μg/g tissue) of injected ‘Valencia’ trees on different rootstocks and with different injection histories 3 to 180 days after the first OTC injection (DAI-1).

Factor	Days after injection							
	Leaves					Roots		
	3 (May 2023)	30 (Jun 2023)	60 (Jul 2023)	90 (Aug 2023)	180 (Nov 2023)	3 (May 2023)	30 (Jun 2023)	60 (Jul 2023)
Infection history								
Late-infected	1.63	0.92	0.59	0.53	0.35	0.000	0.020	0.008
Early-infected	2.82	0.87	0.66	0.63	0.28	0.011	0.056	0.000
*p-value*	*0.0670*	*0.6265*	*0.3937*	*0.4916*	*0.1604*	*0.1346*	*0.1904*	*0.3248*
Rootstock cultivar								
US-812	2.49	0.91	0.63	0.72 a	0.31	0.006	0.016	0.008
US-942	1.97	0.89	0.61	0.43 b	0.32	0.005	0.059	0.000
*p-value*	*0.2382*	*0.8366*	*0.8142*	*0.0481*	*0.7545*	*0.8775*	*0.1359*	*0.3248*
Infection history × Rootstock cultivar								
*p-value*	*0.4543*	*0.2341*	*0.8142*	*0.5736*	*0.8878*	*0.8775*	*0.5056*	*0.3248*
Block								
*p-value*	*0.6358*	*0.1344*	*1.0000*	*1.0000*	*0.7760*	*1.0000*	*0.4279*	*1.0000*

No OTC residues were detectable in the roots 90 and 180 days after injection. Different letters within columns indicate significant differences according to Tukey’s honestly significant difference test. Letters are not shown when P > 0.05.

### 3.3 Tree size and canopy health

**Figure 3:**
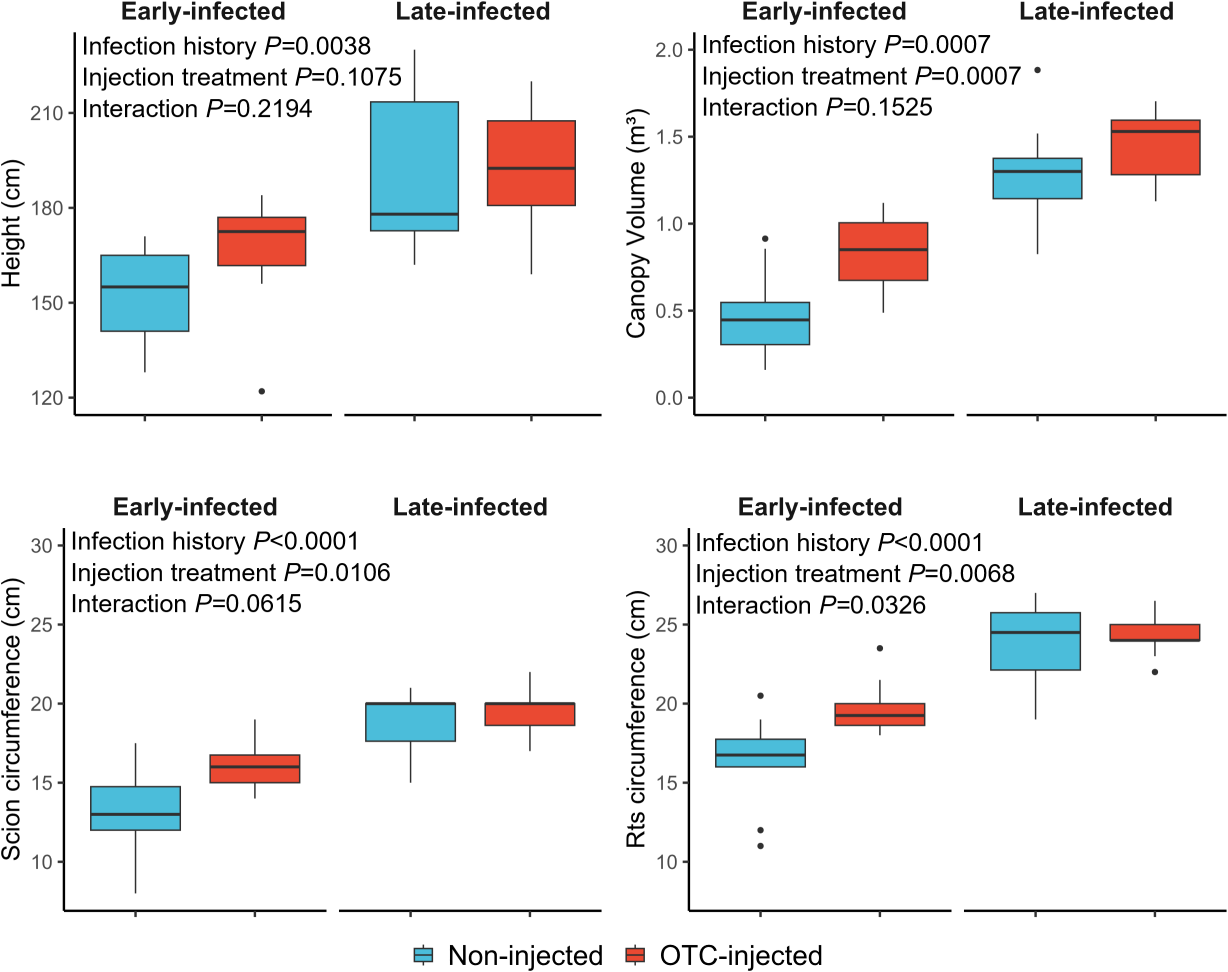
Graphical depiction of tree size responses of non-injected and OTC-injected ‘Valencia’ trees with early and late infection histories in November 2024. For statistical details, see **Table 6**.

In November 2024, late-infected trees were significantly taller (1.93 m) and had a greater scion circumference (19.3 cm), rootstock circumference (24.1 cm), and canopy volume (1.37 m³) than early-infected trees (1.58 m, 14.4 cm, 18.0 cm, and 0.70 m^3^, respectively) (**Table 6**; **Figure 3**). There was no significant effect of rootstock cultivar on any of the growth metrics. Injection treatment significantly affected trunk circumferences and canopy volume, with OTC-injected trees having a greater scion circumference (17.7 cm), rootstock circumference (22.0 cm), and canopy volume (1.14 m³) than non-injected trees (16.0 cm, 20.1 cm, and 0.88 m³, respectively). Block effects were not significant.

**Table 6:** Tree size and health of ‘Valencia’ trees on different rootstocks with different injection histories and injection treatments in November 2024.

Factor	Height (m)	Scion trunk circumf. (cm)	Rootstock trunk circumf. (cm)	Canopy volume (m^3^)	Foliar HLB symptoms	Canopy density	Leaf size (cm^2^)	Chlorophyll content (SPAD)
Infection history								
Late-infected	1.93 a	19.3 a	24.1 a	1.37 a	3.0 b	3.5 a	23.2	68.3
Early-infected	1.58 b	14.4 b	18.0 b	0.70 b	3.3 a	2.7 b	21.4	68.1
*p-value*	*0.0038*	*<0.0001*	*<0.0001*	*0.0007*	*0.0012*	*0.006*	*0.376*	*0.8519*
Rootstock cultivar								
US-812	1.74	16.2	20.5	0.97	3.3 a	2.9	22.2	67.2
US-942	1.77	17.5	21.6	1.05	3.0 b	3.2	22.5	69.2
*p-value*	*0.5557*	*0.0615*	*0.1024*	*0.2203*	*0.0047*	*0.0902*	*0.8411*	*0.0909*
Injection treatment								
OTC-injected	1.80	17.7 a	22.0 a	1.14 a	2.8 b	3.5 a	24.5 a	70.8 a
Non-injected	1.70	16.0 b	20.1 b	0.88 b	3.5 a	2.6 b	20.1 b	65.6 b
*p-value*	*0.1075*	*0.0106*	*0.0068*	*0.0007*	*<0.0001*	*<0.0001*	*0.0138*	*<0.0001*
Infection history × Rootstock cultivar								
*p-value*	*0.1241*	*0.2535*	*0.1367*	*0.2056*	*0.2377*	*0.6714*	*0.191*	*0.1083*
Infection history × Injection treatment								
*p-value*	*0.2194*	*0.0615*	*0.0326*	*0.1525*	*<0.0001*	*0.0324*	*0.3381*	*0.2435*
Rootstock cultivar × Injection treatment								
*p-value*	*0.6779*	*0.8176*	*0.6765*	*0.7563*	*0.2377*	*0.2985*	*0.9784*	*0.2828*
Infection history × Rootstock cultivar × Injection treatment								
*p-value*	*0.2722*	*0.3211*	*0.2454*	*0.927*	*0.1024*	*0.1083*	*0.3122*	*0.3781*
Block								
*p-value*	*0.6915*	*1.0000*	*1.0000*	*0.2408*	*0.2053*	*1.0000*	*1.0000*	*0.2918*

Different letters within columns indicate significant differences according to Tukey’s honestly significant difference test. Letters are not shown when P > 0.05.

**Figure 4:**
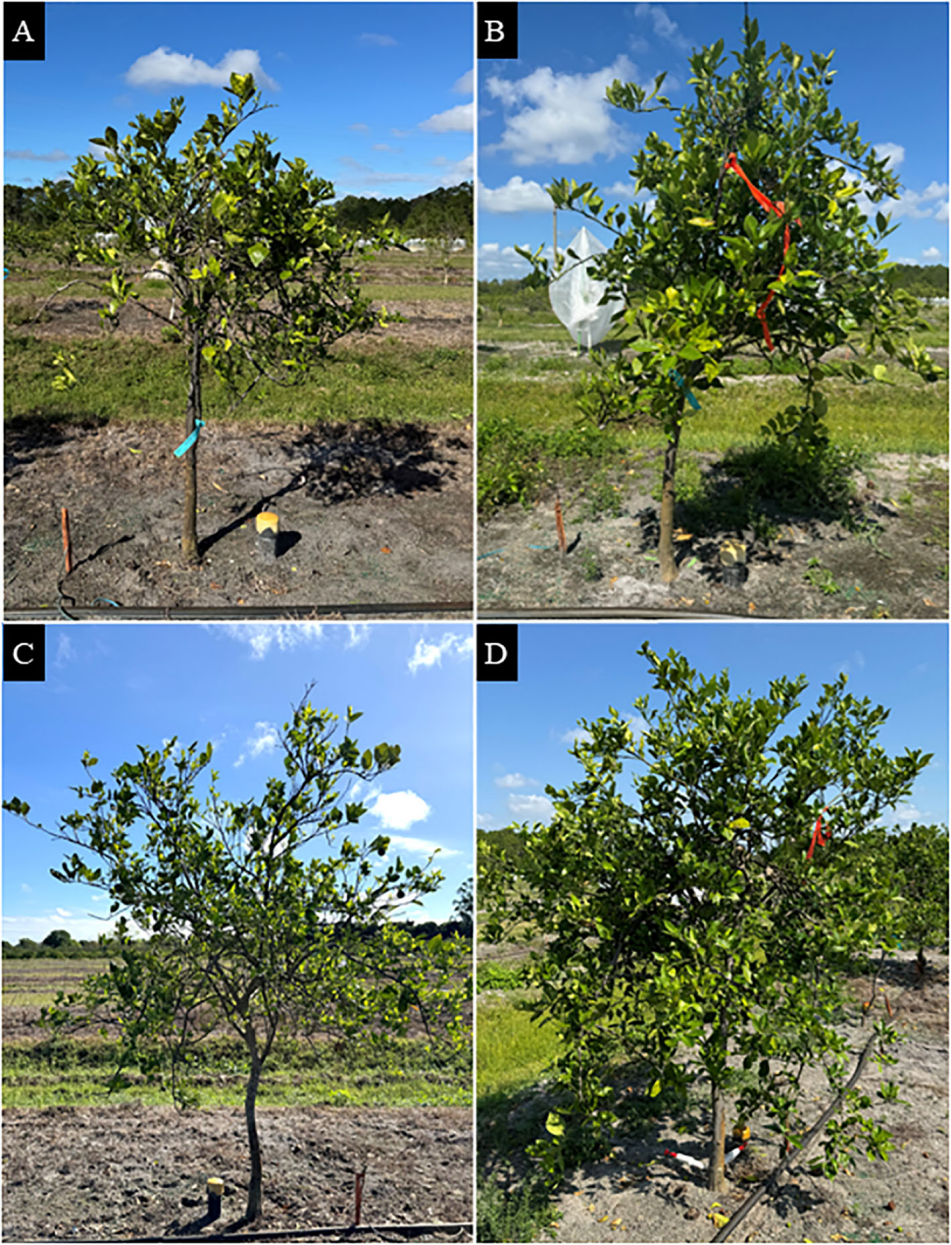
‘Valencia’ sweet orange trees grown in a field site at the Southwest Florida Research and Education Center in Immokalee, FL. **(A)** Early-infected, non-injected tree; **(B)** early-infected, OTC-injected tree; **(C)** late-infected, non-injected tree; and **(D)** late-infected, OTC-injected tree. Photos were taken in March 2025.

Late-infected trees were visually healthier than early infected trees (**Table 6**; **Figure 4**). Late-infected trees had fewer foliar HLB symptoms (3.0 rating) and a denser canopy (3.5 rating) than early-infected trees (3.3 and 2.7 rating, respectively). Trees on US-812 rootstocks had more foliar HLB symptoms (3.3 rating) than trees on US-942 (3.0 rating). Injection treatment significantly affected tree health metrics, with OTC-injected trees showing fewer foliar HLB symptoms (2.8 rating) and denser canopies (3.5 rating) compared to non-injected trees (3.5 and 2.6 rating, respectively). A significant interaction between infection history and injection treatment was observed for foliar HLB symptom ratings and canopy density. Early-infected, non-injected trees exhibited more HLB foliar symptoms and had the smallest canopy density than the other trees (data not shown). Block effects were not significant.

Leaf size was significantly influenced by injection treatment but not by infection history or rootstock cultivar (**Table 6**). OTC-injected trees had significantly larger-size leaves (24.5 cm²) than non-injected trees (20.1 cm²). SPAD readings, representing leaf chlorophyll content, were also significantly higher in OTC-injected trees (70.8) than in non-injected trees (65.6). Infection history and rootstock cultivar had no significant effect on SPAD readings. No significant block effects were observed for the leaf area and SPAD.

### 3.4 Fruit yield, pre-harvest fruit drop, fruit quality, and juice quality

#### 3.4.1 2023–2024 production season

**Figure 5:**
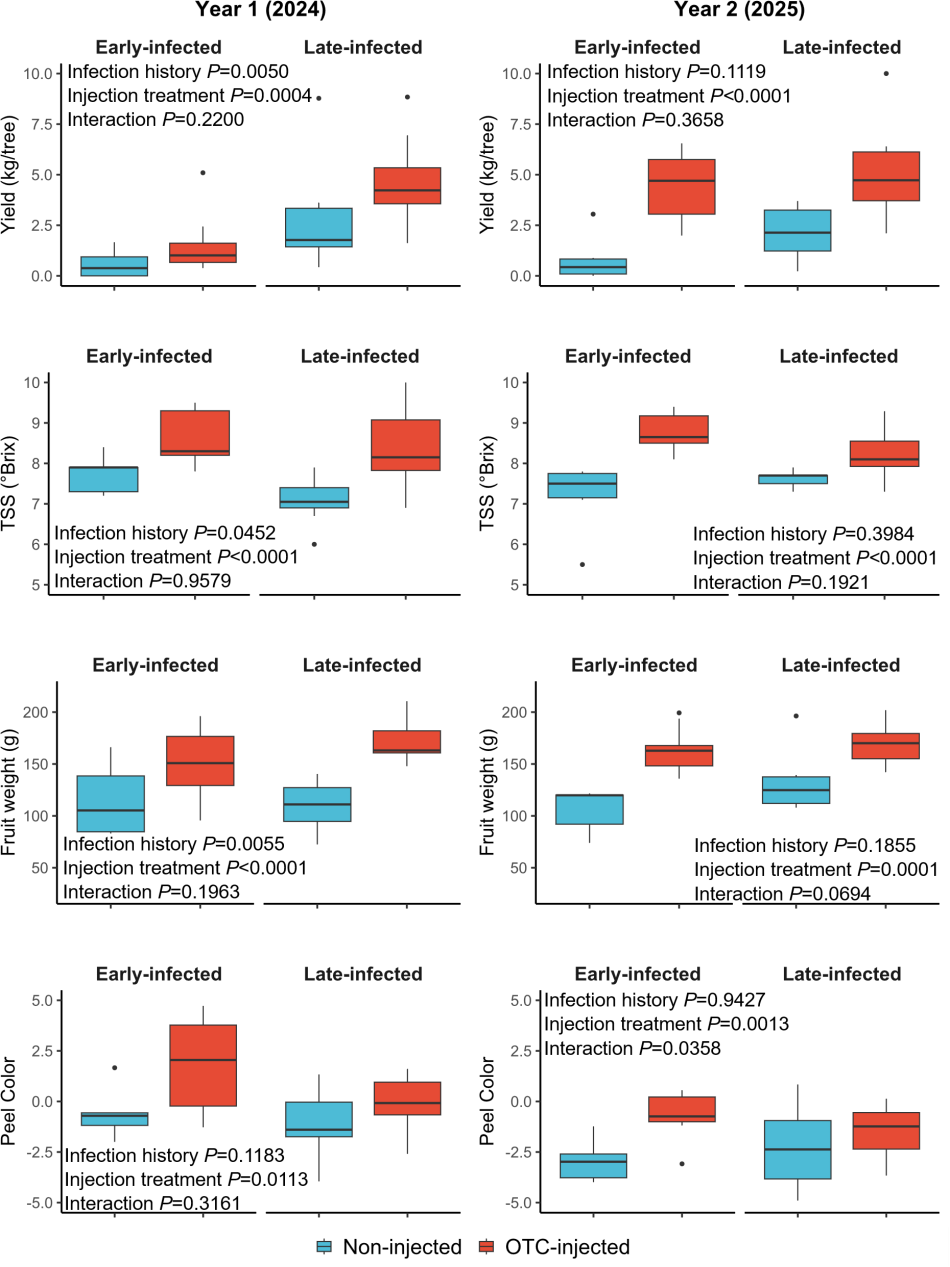
Graphical depiction of selected yield and fruit quality responses of OTC-injected and non-injected ‘Valencia’ trees with early and late infection histories in year 1 (2024) and year 2 (2025). Data are presented as boxplots with median, interquartile range, and outliers.

Both infection history and injection treatment significantly influenced fruit yield in 2024 (**Table 7**; **Figure 5**). Late-infected trees produced significantly more fruits (2.75 kg/tree) than early-infected trees (0.84 kg/tree). Similarly, OTC-injected trees produced more fruits (2.57 kg/tree) than non-injected trees (1.02 kg/tree). Rootstock cultivar had no significant effect on the fruit yield, and there were no significant interactions between infection history, rootstock cultivar, or injection treatment. The percentage of pre-harvest fruit drop was significantly less in late-infected trees (13.0%) compared to early-infected trees (27.9%). Injection treatment also significantly reduced fruit drop, with OTC-injected trees having less drop (4.7%) compared to non-injected trees (36.2%). There were no significant differences in the percentage of fruit drop between rootstock cultivars, and there were no significant interactions.

**Table 7:** Year 1 (2024) yield, percent fruit drop, fruit quality, and juice quality of ‘Valencia’ trees on different rootstocks with different infection histories and injection treatments.

Factor	Yield (kg/tree)	Fruit drop (%)	Fruit size (cm^3^)	Fruit weight (g)	Peel color	TSS	TA (% titratable acid)	TSS/TA
Infection history								
Late-infected	2.75 a	13.0 b	136.7	140.5 a	-0.62	7.7 a	0.69	11.4 b
Early-infected	0.84 b	27.9 a	125.1	104.5 b	0.49	8.2 a	0.60	14.1 a
*p-value*	*0.0050*	*0.0328*	*0.2977*	*0.0055*	*0.1183*	*0.0452*	*0.0538*	*0.0025*
Rootstock cultivar								
US-812	1.95	25.7	134.3	113.4	0.37	8.3 a	0.62	13.9 a
US-942	1.64	15.2	127.5	131.6	-0.49	7.7 b	0.68	11.6 b
*p-value*	*0.3062*	*0.1383*	*0.5207*	*0.1408*	*0.2261*	*0.0125*	*0.2285*	*0.0080*
Injection treatment								
OTC-injected	2.57 a	4.7 b	150.5 a	161.2 a	0.87 a	8.6 a	0.64	13.8 a
Non-injected	1.02 b	36.2 a	111.3 b	83.7 b	-0.99 b	7.3 b	0.65	11.8 b
*p-value*	*0.0004*	*<0.0001*	*0.0010*	*<0.0001*	*0.0113*	*<0.0001*	*0.8678*	*0.0168*
Infection history × Rootstock cultivar								
*p-value*	*0.5964*	*0.0182*	*0.8160*	*0.4112*	*0.4044*	*0.1337*	*0.1159*	*0.2539*
Infection history × Injection treatment								
*p-value*	*0.2200*	*0.0583*	*0.2520*	*0.1963*	*0.3161*	*0.9579*	*0.5072*	*0.4337*
Rootstock cultivar × Injection treatment								
*p-value*	*0.2728*	*0.1187*	*0.3242*	*0.1380*	*0.4889*	*0.0260*	*0.1900*	*0.5303*
Infection history × Rootstock cultivar × Injection treatment								
*p-value*	*0.8817*	*0.0955*	*0.3179*	*0.2750*	*0.4159*	*0.2728*	*0.1728*	*0.1852*
Block								
*p-value*	*0.2478*	*0.2300*	*1.0000*	*1.0000*	*1.0000*	*1.0000*	*1.0000*	*1.0000*

Different letters within columns indicate significant differences according to Tukey’s honestly significant difference test. Letters are not shown when P > 0.05.

OTC-injected trees produced larger fruits (150.5 cm²) than non-injected trees (111.3 cm²) (**Figure 5**), but neither infection history nor rootstock cultivar significantly impacted fruit size (**Table 7**). Fruit weight was significantly greater in late-infected trees (140.5 g) compared to early-infected trees (104.5 g). Similarly, OTC-injected trees produced heavier fruits (161.2 g) than non-injected trees (83.7 g). Rootstock cultivar did not significantly influence fruit weight. Peel color was significantly influenced by injection but not by infection history or rootstock cultivar. OTC-injected trees had a higher peel color index (0.87), meaning they were less green than non-injected trees (-0.99).

Although the TSS content was significantly affected by infection history based on the ANOVA, mean separation was not significant. A significant interaction effect was found between rootstock cultivar and injection treatment. Juice from OTC-injected trees on US-812 had more TSS (9.2°Brix) than juice from injected trees on US-942 (8.0°Brix), non-injected trees on US-812 (7.4°Brix), and non-injected trees on US-942 (7.3°Brix) (data not shown). The TA was not significantly affected by any treatment, but infection history, rootstock cultivar, and injection treatment significantly influenced the TSS/TA ratio. Early-infected trees had a higher ratio (14.1) than late-infected trees (11.4), trees on US-812 had a higher ratio (13.9) than trees on US-942 (11.6), and OTC-injected trees had a higher ratio (13.8) than non-injected trees (11.8). Apart from the interaction between rootstock cultivar and injection treatment for TSS, no significant interactions were observed for any of the other variables. Block effects were also not significant.

#### 3.4.2 2024–2025 production season

Infection history did not significantly affect fruit yield in 2025 (**Table 8**; **Figure 5**). OTC-injected trees produced more fruits (4.80 kg/tree) than non-injected trees (1.42 kg/tree). Rootstock cultivar had no significant effect on fruit yield, and there were no significant interactions between infection history, rootstock cultivar, or injection treatment. Infection history and rootstock cultivar did not influence the percent fruit drop. Injection treatment significantly reduced fruit drop, with OTC-injected trees exhibiting less drop (30.3%) than non-injected trees (45.9%). There were no significant interactions. OTC-injected trees produced significantly larger fruits (141.3 cm^3^) than non-injected trees (109.1 cm^3^) (**Figure 5**), but neither infection history nor rootstock cultivar significantly affected fruit size. OTC-injected trees produced significantly heavier fruits (171.8 g) than non-injected trees (122.3 g), but infection history and rootstock cultivar did not significantly influence fruit weight. Peel color was significantly influenced by injection treatment but not by infection history or rootstock cultivar. OTC-injected trees had a higher peel color index (-1.23), indicating a less green color, than non-injected trees (-3.29).

**Table 8:** Year 2 (2025) yield, percent fruit drop, fruit quality, and juice quality of ‘Valencia’ trees on different rootstocks with different infection histories and injection treatments.

Factor	Yield (kg/tree)	Fruit drop (%)	Fruit size (cm^3^)	Fruit weight (g)	Peel color	TSS	TA (% titratable acid)	TSS/TA
Infection history								
Late-infected	3.66	41.5	128.4	154.6	-2.24	7.8	6.9	11.3
Early-infected	2.57	34.7	122.0	139.5	-2.28	8.0	7.2	11.3
*p-value*	*0.1119*	*0.3677*	*0.4660*	*0.1855*	*0.9427*	*0.3984*	*0.0778*	*0.9061*
Rootstock cultivar								
US-812	2.92	35.5	121.3	146.3	-2.01	7.9	7.1	11.2
US-942	3.30	40.6	129.1	147.7	-2.51	7.8	7.0	11.4
*p-value*	*0.4255*	*0.5015*	*0.4029*	*0.8977*	*0.3840*	*0.8408*	*0.9288*	*0.6365*
Injection treatment								
OTC-injected	4.80 a	30.3 b	141.3 a	171.8 a	-1.23 a	8.5 a	6.5 b	13.0 a
Non-injected	1.42 b	45.9 a	109.1 b	122.3 b	-3.29 b	7.3 b	7.6 a	9.7 b
*p-value*	*<0.0001*	*0.0460*	*0.0012*	*0.0001*	*0.0013*	*<0.0001*	*<0.0001*	*<0.0001*
Infection history × Rootstock cultivar								
*p-value*	*0.5180*	*0.2024*	*0.2329*	*0.8882*	*0.0639*	*0.6238*	*0.1761*	*0.3946*
Infection history × Injection treatment								
*p-value*	*0.3658*	*0.7431*	*0.2975*	*0.0694*	*0.0358*	*0.1921*	*0.2496*	*0.0869*
Rootstock cultivar × Injection treatment								
*p-value*	*0.7693*	*0.9111*	*0.8826*	*0.2582*	*0.0734*	*0.0337*	*0.1916*	*0.5839*
Infection history × Rootstock cultivar × Injection treatment								
*p-value*	*0.3199*	*0.8467*	*0.3009*	*0.5242*	*0.2838*	*0.9052*	*0.0038*	*0.0598*
Block								
*p-value*	*0.6868*	*1.0000*	*0.2970*	*1.0000*	*1.0000*	*1.0000*	*1.0000*	*1.0000*

Different letters within columns indicate significant differences according to Tukey’s honestly significant difference test. Letters are not shown when P > 0.05.

Juice from OTC-injected trees had significantly more TSS (8.5°Brix) compared to non-injected trees (7.3°Brix) (**Table 8**). Infection history and rootstock cultivar did not significantly affect TSS. The TA was significantly lower in OTC-injected trees (6.5%) compared to non-injected trees (7.6%), while infection history and rootstock cultivar did not significantly affect TA. The TSS/TA ratio was significantly influenced by injection treatment, with OTC-injected trees exhibiting a higher ratio (13.0) than non-injected trees (9.7), while neither infection history nor rootstock cultivar significantly influenced the TSS/TA ratio.

No significant interactions were observed between infection history, rootstock cultivar, or injection treatment for most variables. However, a significant interaction between rootstock cultivar and injection treatment was observed for TSS. Juice from OTC-injected trees on US-812 had more TSS (9.1°Brix) than juice from non-injected trees on U-812 and US-942 (7.0°Brix and 7.4°Brix, respectively) (data not shown). A significant interaction between infection history and injection treatment was also observed for peel color. Late- and early-infected, OTC-injected trees had a less green peel color (-0.1) than early-infected, non-injected trees (-5.0) (**Figure 4**). Block effects were not significant.

### 3.5 Leaf nutrient content

There were no significant effects of infection history and injection treatment on the leaf macro- and micronutrient content in July 2023 (**Supplementary Table S4**). In contrast, the rootstock cultivar significantly impacted some of the leaf macro- and micronutrients. US-942 induced higher levels of N (2.1%) and P (0.26%) compared to US-812 (2.0% and 0.23%, respectively). A higher Mg and Zn content was induced by US-812 (0.31% and 24.4 ppm, respectively) than by US-942 (0.28% and 16.2 ppm, respectively). None of the other macro- and micronutrients were significantly affected by rootstock cultivar. There was a significant interaction between infection history and injection treatment for Mn levels, but there was no significant mean separation (data not shown). No other significant interactions or block effects were observed.

There were no significant effects of infection history, rootstock cultivar, or injection treatment on the leaf macro- and micronutrient content in July 2024 (**Supplementary Table S5**). There were no significant block effects or interactions, except between infection history, rootstock cultivar, and injection for Fe, though there was no significant mean separation (data not shown).

### 3.6 Multivariate (PCA) analysis

**Figure 6:**
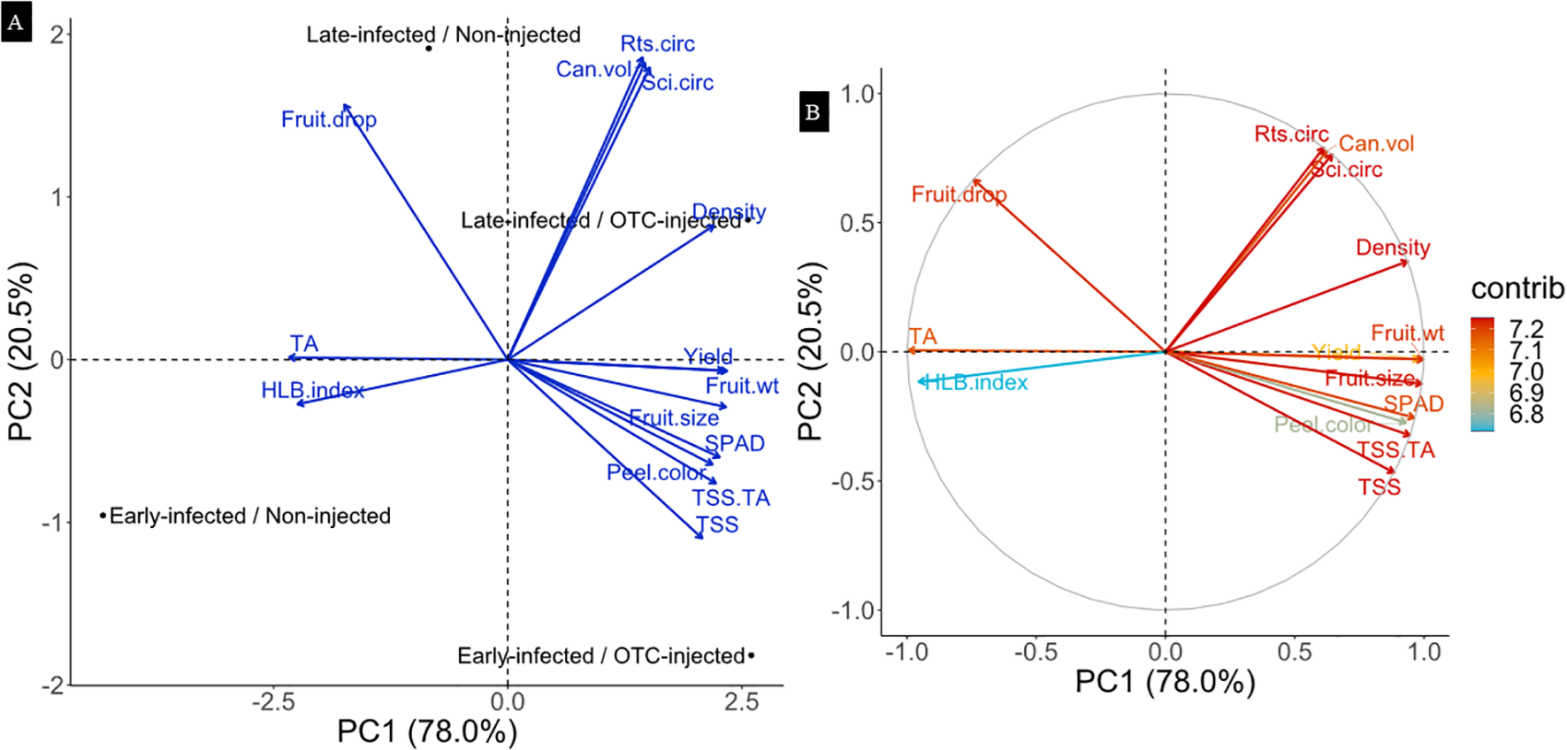
Principal component analysis (PCA) biplots representing the relationships between infection history and injection treatment with tree growth, yield, and physiological response variables after two years of OTC injection. **(A)**: PCA biplot. **(B)**: Correlation plot indicating the contributions of each response variable. The length and direction of the arrows represent the strength and direction of correlation for each trait. The grey (correlation) circle indicates the maximum possible contribution of variables to the PCA axes, with arrows closer to the edge showing stronger representation in the two-dimensional PCA.

Principal component analysis (PCA) was conducted, excluding rootstock cultivar as a factor. The PCA plot shows that the first two principal components explained 98.5% of the total variance, with PC1 accounting for 78.0% and PC2 for 20.5% (**Figure 6A**). Along PC1, separation occurred mainly between OTC-injected and non-injected trees. This separation was mainly associated with yield, fruit weight, fruit size, peel color, and SPAD, which were positively correlated with injection, while HLB index and TA values were negatively correlated with injection. Along PC2, variation was primarily explained by infection history. Canopy volume and rootstock, and scion circumference contributed most to that separation and were positively correlated with late infection. Contribution analysis showed that all variables contributed similarly to PC1 and PC2, with values ranging from 6.8% to 7.2% (**Figure 6B**).

### 3.7 Root endorhizosphere bacterial community

**Figure 7:**
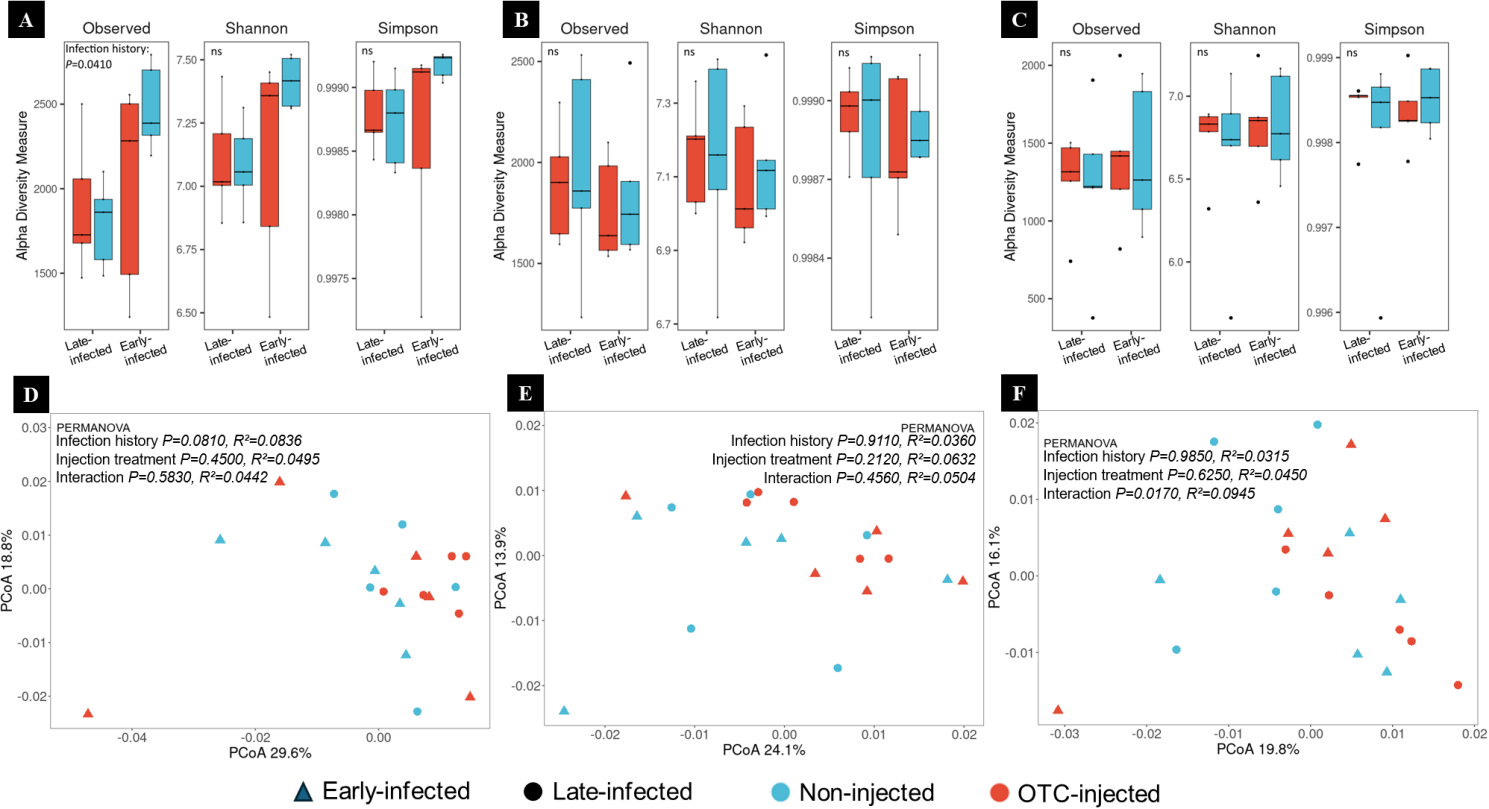
**(A–C)** Alpha diversity of root endorhizosphere bacterial communities of ‘Valencia’/US-942 trees with different infection histories and injection treatments at 30 **(A)**, 90 **(B)**, and 270 days **(C)** after the first injection 1 (DAI-1). Boxplots display observed richness, Shannon, and Simpson diversity indices of bacterial communities in OTC-injected (red) and non-injected (blue) trees with late and early infection histories. Statistical comparisons are annotated with “ns” for non-significant and “*” for significant differences (p < 0.05). **(D–F)** Beta diversity of root endorhizosphere bacterial communities at 30 **(D)**, 90 **(E)**, and 270 **(F)** DAI-1. Principal Coordinates Analysis (PCoA) plots are based on Bray-Curtis dissimilarities.

There was no significant effect of injection treatment on the bacterial diversity and community structure 30 days after injection 1 (30 DAI-1) (**Figure 7A**). However, there was a significant effect of the infection history. The observed richness of bacterial communities was lower in late-infected compared to early-infected trees, but Shannon and Simpson diversity indices revealed no significant differences. PCoA based on Bray-Curtis dissimilarities showed that the first two principal components explained 48.4% of the total variance, with 29.6% attributed to PCoA1 and 18.8% to PCoA2 (**Figure 7D**). PERMANOVA analysis showed no significant effects of infection history, injection treatment, or their interaction.

There was no significant effect of injection treatment or injection history on the bacterial diversity and community structure at 60 DAI-1 (**Figure 7B**). PCoA based on Bray-Curtis dissimilarities revealed that the first two principal components explained 38% of the total variance, with 24.1% attributed to PCoA1 and 13.9% to PCoA2 (**Figure 7E**). PERMANOVA analysis showed no significant effects of infection history, injection treatment, or their interaction.

There was no significant effect of injection treatment or injection history on the bacterial diversity and community structure at 270 DAI-1. (**Figure 7C**). PCoA based on Bray-Curtis dissimilarities, revealed that the first two principal components accounted for 35.9% of the total variance, with 19.8% attributed to PCoA1 and 16.1% to PCoA2 (**Figure 7F**). PERMANOVA analysis indicated no significant effects for infection history or injection treatment. However, a significant interaction effect was observed.

## 4 Discussion

This research builds upon existing approaches to HLB management by integrating preventative and therapeutic strategies applicable to new plantings and mature orchards. The efficacy of using IPCs to protect young trees from ACP infestation and *C*Las infection was previously demonstrated by Gaire et al. (2023) and Tardivo et al. (2024). IPCs are an effective physical barrier during the critical early growth stages, allowing trees to establish a robust root and canopy system before becoming exposed to the pathogen. Our findings expand on this by combining the benefits of preventing infection during the tree establishment phase with curative measures, i.e., the systemic delivery of OTC upon first-time infection.

### 4.1 OTC trunk injection following IPC removal reduces *C*Las titers

Late-infected trees, i.e. trees that were protected from psyllids by IPCs for 18 months, retained lower bacterial titers than early-infected trees for the first five months after IPC removal and remained visually healthy, indicating a lasting benefit. This reinforces the role of IPCs as an important component of an integrated HLB management program. Trunk injection of OTC significantly reduced leaf *C*Las titers in both early- and late-infected trees, confirming the efficacy of systemically delivered OTC as previously observed (Hu and Wang, 2016; Archer et al., 2022a, 2023; Castellano-Hinojosa et al., 2024). OTC-injected trees consistently exhibited higher leaf Ct-values (lower bacterial titers) than non-injected trees, with effects persisting for several months after injection. This aligns with the OTC residues, which were detectable in the leaves for up to 180 days post-injection. Archer et al. (2022a) and Archer and Albrecht (2023) reported similar lasting reductions in bacterial titers in sweet orange trees growing under HLB-endemic conditions.

However, this study did not show any efficacy of the OTC in reducing root *C*Las titers, contrary to previous studies (Archer et al., 2022a; Castellano-Hinojosa et al., 2024). In fact, fibrous roots of OTC-injected trees exhibited lower Ct-values intermittently, indicating higher *C*Las titers compared to non-injected trees. This may be associated with the OTC-induced improved tree health, resulting in a higher fibrous root growth intensity in response to the OTC injection and, therefore, a higher translocation rate of the bacteria to the growing roots according to source-to-sink relationships (Pulici et al., 2023). A similar effect was observed for the fibrous roots of late-infected trees. However, fibrous root bacterial titers were considerably lower than leaf bacterial titers as previously reported (Tatineni et al., 2008; Tardivo et al., 2023).

There were noticeable differences in efficacy between the first and second year of OTC injections. In the first year, the largest differences in Ct-values were observed up to 90 days after injection. This effect was more pronounced in the second year, lasting until 180 days after injection. This suggests a cumulative impact of repeated OTC injections, though it may also be attributed to the higher OTC dose administered in year 2, along with a better distribution of the antibiotic within the tree stemming from the two-sided injection.

The loss of bactericide efficacy over time underscores the importance of optimizing injection timing and dosage. Maintaining effective bactericide concentrations in target tissues is necessary to prevent re-infection, but the cost of multiple injections per year and the risks from trunk damage associated with the injections (Van Vuuren, 1977; Archer and Albrecht, 2023) are likely prohibitive. As it is unlikely that one annual OTC injection can completely eradicate *C*Las from heavily infected trees, controlling psyllid populations as part of an integrated management program remains essential to cope with HLB (De Carli et al., 2018; Bassanezi et al., 2020, 2024).

### 4.2 IPCs and OTC trunk injections improve tree growth and productivity

The preventative and therapeutic effects of IPCs and systemically delivered OTC, respectively, were evident in the improved vegetative performance of late-infected (previously IPC-protected) and OTC-injected trees, as shown by the larger canopy volume, higher canopy density, greener canopy color, and larger leaf size. Although leaf and canopy health often correlate with nutrient availability (Spann and Schumann, 2009; Neupane et al., 2023), our study found no significant changes in leaf nutrient contents due to injection treatment or infection history, and leaf nutrients were in the adequate range for citrus (Morgan and Kadyampakeni, 2020). Regular applications of slow-release fertilizer during the study may have been responsible for the lack of a measurable effect. While enhanced nutrient management alone has proven ineffective in slowing HLB progression (Bassanezi et al., 2021; 2024), it remains essential for sustaining tree health and productivity in infected orchards (Morgan and Kadyampakeni, 2020).

Delaying *C*Las infection through use of IPCs also resulted in lasting benefits in tree size and canopy volume regardless of injection. Even 28 months after IPC removal, late-infected trees remained taller, with larger trunk diameters and larger, denser canopies. Similarly, Gaire et al. (2024) reported that IPC-protected trees maintained superior growth metrics relative to unprotected trees in HLB-endemic conditions. The increased trunk size of late-infected trees enables earlier OTC injections, allowing earlier therapeutic intervention and therefore preventing *C*Las from accumulating to the high levels leading to HLB-induced tree decline.

Improved tree health translated into significantly higher productivity regardless of the rootstock, particularly in the second year of the study. In the 2023–2024 production season, OTC-injected trees produced more fruits compared to non-injected trees, and this difference was even more pronounced in 2024–2025 after two consecutive years of injections. Other studies also reported significant increases in yield and juice quality in different citrus cultivars following trunk injection of OTC using various injection approaches (Hu and Wang, 2016; Hu et al., 2018; Archer and Albrecht, 2023; Archer et al., 2023; Albrecht et al., 2025a). Earlier studies in other countries documented foliar and fruit symptom remediation after trunk injection of antibiotics but yield and other tree responses were not reported (Schwarz et al., 1974; Da Graça, 1991). It is important to note that external stressors such as Hurricane Milton in late 2024 and rust mite infestations during the 2024–2025 season may have limited the yield potential of the trees in this study, potentially underestimating the full benefits of OTC injections on production.

Trees exhibiting more severe HLB symptoms generally experience significantly more fruit drop than trees with less severe symptoms (Tang et al., 2019 and 2020). In our study, pre-harvest fruit drop was reduced in OTC-treated trees across both seasons, consistent with these findings. Beyond the effects of injection treatment, infection history also significantly influenced fruit drop, but only during the first year of injection, when late-infected trees exhibited lower drop rates than early-infected trees, indicating a stronger influence of injection treatment over time following IPC removal.

Studies with other scion/rootstock combinations have found similar benefits on productivity in response to OTC trunk injections (Archer et al., 2023; Albrecht et al., 2025b; Worbington et al., 2025) although the responses varied among studies. Some of these variations are likely associated with differences in production conditions, OTC dose, tree age, tree health status at the time of injections, and other factors. For specialty varieties like grapefruits and mandarins, more research is needed to assess the economic potential of OTC injection therapy as well as the applicability of using IPCs. For example, a recent study testing different mandarins under IPCs found that not all varieties are equally suited for this management strategy (Ben-Abdallah et al., in press).

### 4.3 OTC trunk injection improves fruit and juice quality

*C*Las infection severely impairs fruit development, resulting in smaller, asymmetrical fruit with poor peel coloration and altered flavor profiles, including increased bitterness and off-flavor volatiles (Baldwin et al., 2010; Rosales and Burns, 2011; Dala-Paula et al., 2019). In our study, OTC-injected trees consistently produced larger and heavier fruits across both seasons, confirming prior findings that demonstrated that improved tree health and canopy density support better fruit development (Archer et al., 2023). These effects were especially pronounced in the 2024–2025 production season. Peel color was also significantly improved in OTC-injected trees. These improvements of external quality traits suggest that trunk injections of OTC can benefit fresh fruit markets, where external fruit quality is essential.

Internal fruit quality also improved significantly in response to OTC injections. In both years, juice from OTC-injected trees contained more total soluble solids (TSS) and had a higher TSS/TA ratio, with stronger effects observed in year 2, reinforcing the benefits of consecutive injections. These internal fruit quality improvements are particularly relevant in Florida’s juice market, where growers are compensated based on TSS (USDA-NASS, 2024). Our findings are consistent with earlier reports documenting enhanced juice quality following OTC trunk injection (Hu et al., 2018; Archer et al., 2023; Albrecht et al., 2025a). The strong positive association of fruit quality attributes and OTC injection was evident in the multivariate (PCA) analysis, which showed a strong separation between injected and non-injected trees, driven mainly by fruit quality responses, although other tree responses also contributed.

The rootstock cultivar also influenced juice quality to some extent. The two rootstocks used in this study, US-812 and US-942, share a similar genetic background. Both are citrandarins, i.e., they are derived from a cross between mandarin (*Citrus reticulata*) and trifoliate orange (*Poncirus trifoliata*), which may explain their comparable field performance. Citrandarins are generally reported to perform well under HLB pressure in addition to inducing other favorable attributes to the grafted tree (Boava et al., 2015; Girardi et al., 2021), and both US-812 and US-942 have been among the most propagated rootstocks in Florida for many years (FDACS, 2024). However, in the 2023–2024 production season, trees on US-942 produced significantly less TSS and a lower TSS/TA ratio than US-812. This trend is consistent with findings from other ongoing trials in our program, where US-942 induces inferior juice quality compared with other rootstocks. Despite rootstock effects on some of the measured variables, responses to OTC injection were similar for both cultivars. Other studies with different scion/rootstock combinations also documented significant improvements in fruit and juice quality after OTC trunk injection (Archer et al., 2023; Albrecht et al., 2025b; Worbington et al., 2025).

### 4.4 Systemic OTC delivery has no lasting effect on the active root bacterial community

OTC injections resulted in little change to the root endorhizosphere bacterial abundance and diversity. The stability or recovery of the root endorhizosphere bacterial community following the injections suggests a minimal impact of this treatment. It must be noted that only US-942 roots were used in this analysis, suggesting that our results may not be applicable to other rootstock cultivars used in commercial citrus production. Across all sampling time points, diversity metrics, including observed richness, Shannon, and Simpson diversity indices, revealed no significant differences between treatments, except for the observed richness of bacterial communities at 30 DAI-1, which was higher in early-infected compared to late-infected trees. This may be due to the proliferation of opportunistic bacteria in the declining fibrous roots of early-infected trees. The lack of clustering in PCoA analyses and the lack of statistical significance of the PERMANOVA further support the lack of disruption to the root-associated bacterial community by OTC injections. These findings are consistent with a recent study demonstrating that localized antibiotic applications cause few disruptions to non-target rhizosphere microbial communities (Castellano-Hinojosa et al., 2024)​. Another study, which investigated the effect of trunk-applied penicillin in grapefruit trees grafted on Swingle rootstock, revealed that despite altering the abundance of certain taxa, the antibiotic did not cause overall shifts in rhizosphere or endophytic community structure, as indicated by the lack of clustering in PCoA (Ascunce et al., 2019). This further supports the concept that antibiotic effects are localized and do not cause any long-term destabilizing effects on the root bacterial communities when they are delivered directly into the tree vascular system. The absence of detectable OTC residues in the fibrous roots further supports this notion. This is also consistent with prior studies showing limited movement of OTC into root tissues (Hijaz et al., 2020). As highlighted by Wise et al. (2014), unlike foliar sprays, which can result in spray drift and broad environmental exposure, trunk injections deliver pesticides directly into the vascular system, limiting their dispersal into the surrounding soil and reducing unintended ecological consequences.

## 5 Conclusion

This study highlights the benefit of integrating use of IPCs with OTC trunk injections to delay the onset and improve the management of HLB in young sweet orange trees. IPCs serve as an effective preventative strategy during the critical tree establishment phase and confer lasting benefits to tree growth even after their removal. Trunk injection of OTC is a curative measure, suppressing bacterial titers and supporting tree health, productivity, and fruit and juice quality once trees become infected. These complementary strategies expand the viable management options for Florida citrus growers and offer a model for other affected citrus production regions. Importantly, OTC injections did not disrupt the root endorhizosphere bacterial community, underscoring the treatment’s precision and minimal ecological impact when applied via trunk injection.

## Data Availability

The data presented in the study are deposited in the NCBI repository, accession number BioProject: PRJNA1291227.

## Supplementary material

Supplementary Table 1 Interaction effects for Ct-values of leaves and fibrous roots from ‘Valencia’ trees on different rootstocks with different infection histories and injection treatments at different days after the first (DAI-1) and second OTC injection (DAI-2). Different letters within columns indicate significant differences according to Tukey’s honestly significant difference test. Letters are not shown when P > 0.05.

Supplementary Table 2 Ct-values of fibrous roots of ‘Valencia’ trees on different rootstocks with different infection histories and injection treatments 3 to 360 days after the first OTC injection (DAI-1). Different letters within columns indicate significant differences according to Tukey’s honestly significant difference test. Letters are not shown when P > 0.05.

Supplementary Table 3 Ct-values of fibrous roots of ‘Valencia’ trees on different rootstocks with different infection histories and injection treatments 3 to 360 days after the second OTC injection (DAI-2). Different letters within columns indicate significant differences according to Tukey’s honestly significant difference test. Letters are not shown when P > 0.05.

Supplementary Table 4 Macro and micronutrient content of leaves from ‘Valencia’ trees on different rootstocks with different infection histories and injection treatments in July 2023. Different letters within columns indicate significant differences according to Tukey’s honestly significant difference test. Letters are not shown when P > 0.05.

Supplementary Table 5 Macro and micronutrient content of leaves from ‘Valencia’ trees on different rootstocks with different infection histories and injection treatments in July 2024. Different letters within columns indicate significant differences according to Tukey’s honestly significant difference test. Letters are not shown when P > 0.05.
