# Supplementary material for: Delaying Candidatus Liberibacter asiaticus infection of citrus trees through use of individual protective covers and systemic delivery of oxytetracycline
Source: Front Plant Sci. 2025 Oct 28;16:1671217. doi: 10.3389/fpls.2025.1671217 (PMC12602398; doi:10.3389/fpls.2025.1671217)
Supplement: Supplementary Table 1 — Interaction effects for Ct-values of leaves and fibrous roots from ‘Valencia’ trees on different rootstocks with different infection histories and injection treatments at different days after the first (DAI-1) and second OTC injection (DAI-2). Different letters within columns indicate significant differences according to Tukey’s honestly significant difference test. Letters are not shown when P > 0.05. [file DataSheet1.pdf]

Supplementary Table 1. Interaction effects for Ct-values of leaves and fibrous roots from 'Valencia' trees on different rootstocks with different infection histories and injection treatments at different days after the first (DAI-1) and second OTC injection (DAI-2).

Factor	Leaves		Roots		Leaves	
	30 DAI-1 (Jun 2023)	360 DAI-1 (May 2024)	90 DAI-1 (Aug 2023)	90 DAI-2 (Aug 2024)	270 DAI-2 (Feb 2025)	360 DAI-2 (May 2025)
Infection history × Rootstock cultivar						
Late-infected × US-812	21.9	22.2	31.5	27.1	24.3	23.6
Early-infected × US-812	21.9	22.4	36.9	24.9	24.4	23.7
Late-infected × US-942	23.1	22.0	35.0	23.9	23.5	22.7
Early-infected × US-942	22.1	23.5	34.1	23.6	25.6	23.0
p-value	*0.3935*	*0.0821*	*0.0129*	*0.4336*	*0.0982*	*0.0821*
Infection history × Injection treatment						
Late-infected × OTC-injected	22.2 ab	22.1 b	32.1	28.5	24.3 b	23.6 a
Late-infected × Non-injected	22.8 ab	22.0 b	34.4	22.5	23.5 b	23.7 a
Early-infected × OTC-injected	23.8 a	23.8 a	35.6	27.5	26.9 a	22.9 a
Early-infected × Non-injected	20.2 b	22.2 b	35.4	21.0	23.2 b	23.8 a
p-value	*0.0004*	*0.0446*	*0.2819*	*0.8224*	*0.0203*	*0.0446*
Rootstock cultivar × Injection treatment						
US-812 × OTC-injected	23.1	22.4	32.9	30.3 a	25.4	24.3
US-812 × Non-injected	20.7	22.2	35.5	21.7 b	23.3	23.1
US-942 × OTC-injected	22.9	23.5	34.7	25.7 b	25.7	22.2
US-942 × Non-injected	22.2	22.0	34.3	21.8 b	23.4	23.5
p-value	*0.1386*	*0.0559*	*0.2218*	*0.0439*	*0.8544*	*0.0559*
Infection history × Rootstock cultivar × Injection treatment						
Late-infected × US-812 × OTC-injected	22.5	22.2 b	30.0	31.4	24.8	24.3 a
Late-infected × US-812 × Non-injected	21.3	22.1 b	33.1	22.7	23.7	23.0 a
Late-infected × US-942 × OTC-injected	21.9	22.0 b	34.2	25.5	23.7	22.9 a
Late-infected × US-942 × Non-injected	24.2	22.0 b	35.8	22.3	23.3	22.5 a
Early-infected × US-812 × OTC-injected	23.6	22.5 b	35.9	29.1	26.0	24.3 a
Early-infected × US-812 × Non-injected	20.1	22.4 b	37.9	20.7	22.8	23.1 a
Early-infected × US-942 × OTC-injected	24.0	25.0 a	35.3	25.8	27.8	21.5 a
Early-infected × US-942 × Non-injected	20.2	22.0 b	32.9	21.3	23.5	24.5 a
p-value	*0.0887*	*0.0404*	*0.5425*	*0.7526*	*0.4624*	*0.0404*
Block						
p-value	*0.7677*	*1.0000*	*1.0000*	*0.3364*	*1.0000*	*1.0000*

Different letters within columns indicate significant differences according to Tukey's honestly significant difference test. Letters are not shown when P > 0.05.

Supplementary Table 2. Ct-values of fibrous roots of 'Valencia' trees on different rootstocks with different infection histories and injection treatments 3 to 360 days after the first OTC injection (DAI-1).

Factor	Days after injection						
	3 (May 2023)	30 (Jun 2023)	60 (Jul 2023)	90 (Aug 2023)	180 (Nov 2023)	270 (Feb 2024)	360 (May 2024)
Infection history							
Late-infected	30.6 b	31.8	33.6 b	33.2	34.6	33.0	34.9
Early-infected	34.1 a	34.1	36.2 a	35.5	35.4	33.0	36.4
p-value	*0.0010*	*0.2782*	*0.0172*	*0.0657*	*0.5592*	*0.9471*	*0.2266*
Rootstock cultivar							
US-812	32.8	33.5	34.5	34.2	36.3 a	33.7	36.4
US-942	31.8	32.4	35.3	34.5	33.8 b	32.2	35.0
p-value	*0.6257*	*0.1654*	*0.3586*	*0.7928*	*0.0451*	*0.2567*	*0.1208*
Injection treatment							
OTC-injected	32.3	31.6 b	34.1	33.8	34.4	31.6 b	35.4
Non-injected	32.4	34.1 a	35.6	34.9	35.6	34.3 a	35.9
p-value	*0.9223*	*0.0043*	*0.1110*	*0.3802*	*0.2980*	*0.0433*	*0.5606*
Infection history× Rootstock cultivar							
p-value	*0.8453*	*0.3137*	*0.6425*	*0.0129*	*0.3182*	*0.6338*	*0.4957*
Infection history × Injection treatment							
p-value	*0.8263*	*0.0001*	*0.9082*	*0.2819*	*0.2771*	*0.9594*	*0.3403*
Rootstock cultivar × Injection treatment							
p-value	*0.6421*	*0.1240*	*0.2887*	*0.2218*	*0.9964*	*0.7678*	*0.1168*
Infection history × Rootstock cultivar × Injection treatment							
p-value	*0.5912*	*0.0521*	*0.5974*	*0.5425*	*0.7937*	*0.5906*	*0.1941*
Block							
p-value	*1.0000*	*0.7006*	*1.0000*	*1.0000*	*1.0000*	*1.0000*	*1.0000*

Different letters within columns indicate significant differences according to Tukey's honestly significant difference test. Letters are not shown when P > 0.05.

Supplementary Table 3. Ct-values of fibrous roots of ‘Valencia’ trees on different rootstocks with different infection histories and injection treatments 3 to 360 days after the second OTC injection (DAI-2).

Factor	Days after injection						
	3 (May 2024)	30 (Jun 2024)	60 (Jul 2024)	90 (Aug 2024)	180 (Nov 2024)	270 (Feb 2025)	360 (May 2025)
Infection history							
Late-infected	36.1	32.8	34.2	35.1	34.8	32.6	32.1
Early-infected	35.1	34.1	33.5	34.6	34.2	33.2	31.4
p-value	*0.3596*	*0.1911*	*0.5592*	*0.6706*	*0.5814*	*0.6890*	*0.5491*
Rootstock cultivar							
US-812	34.7	34.0	33.9	35.0	35.7 a	33.3	31.2
US-942	36.4	33.0	33.8	34.8	33.4 b	32.5	32.3
p-value	*0.1136*	*0.2260*	*0.8775*	*0.8848*	*0.0412*	*0.5400*	*0.2199*
Injection treatment							
OTC-injected	35.3	33.0	33.5	34.4	34.4	31.9	31.9
Non-injected	35.9	34.0	34.2	35.4	34.7	33.9	31.7
p-value	*0.5747*	*0.1995*	*0.4036*	*0.2948*	*0.7825*	*0.1316*	*0.7686*
Infection history × Rootstock cultivar							
p-value	*0.3830*	*0.6859*	*0.6001*	*0.7771*	*0.6865*	*0.9986*	*0.1163*
Infection history × Injection treatment							
p-value	*0.4511*	*0.4369*	*0.4445*	*0.1561*	*0.9162*	*0.9643*	*0.3910*
Rootstock cultivar × Injection treatment							
p-value	*0.6920*	*0.1075*	*0.1384*	*0.2690*	*0.4180*	*0.9138*	*0.1758*
Infection history × Rootstock cultivar × Injection treatment							
p-value	*0.5512*	*0.4382*	*0.9025*	*0.8969*	*0.4089*	*0.2390*	*0.4461*
Block							
p-value	*1.0000*	*0.5940*	*0.8481*	*0.2653*	*0.5525*	*1.0000*	*0.9710*

Different letters within columns indicate significant differences according to Tukey’s honestly significant difference test. Letters are not shown when $P > 0.05$.

Supplementary Table 4. Macro and micronutrient content of leaves from 'Valencia' trees on different rootstocks with different infection histories and injection treatments in July 2023.

Factor	N (%)	P (%)	K (%)	Ca (%)	Mg (%)	S (%)	B (ppm)	Zn (ppm)	Mn (ppm)	Fe (ppm)	Cu (ppm)
Infection history											
Late-infected	2.03	0.24	1.21	3.60	0.29	0.23	102.2	20.9	113.6	79.4	33.0
Early-infected	2.03	0.24	1.21	3.63	0.30	0.23	100.6	20.6	110.6	71.7	31.0
p-value	*0.9536*	*0.4841*	*0.9015*	*0.7218*	*0.7659*	*0.5953*	*0.6561*	*0.9296*	*0.5579*	*0.0634*	*0.4116*
Rootstock cultivar											
US-812	2.0 b	0.23 b	1.22	3.60	0.31 a	0.22	98.6	25.4 a	109.4	74.8	34.3
US-942	2.1 a	0.26 a	1.21	3.63	0.28 b	0.23	104.2	16.2 b	114.8	76.2	29.7
p-value	*0.0427*	*0.0004*	*0.7509*	*0.8295*	*0.0146*	*0.1913*	*0.1717*	*0.0336*	*0.3446*	*0.7537*	*0.0756*
Injection treatment											
OTC-injected	2.04	0.25	1.23	3.68	0.30	0.23	102.0	22.5	111.6	75.8	33.6
Non-injected	2.03	0.24	1.20	3.55	0.29	0.22	100.8	19.0	112.7	75.3	30.4
p-value	*0.8071*	*0.3935*	*0.5058*	*0.2427*	*0.2173*	*0.1185*	*0.7560*	*0.3868*	*0.8294*	*0.9008*	*0.1759*
Infection history × Rootstock cultivar											
p-value	*0.2844*	*0.4841*	*0.5704*	*0.7077*	*0.5927*	*0.7496*	*0.9892*	*0.8895*	*0.3128*	*0.6015*	*0.5130*
Infection history × Injection treatment											
p-value	*0.1298*	*0.6966*	*0.4903*	*0.6253*	*0.7659*	*0.4583*	*0.0549*	*0.3109*	*0.0033*	*0.9602*	*0.9831*
Rootstock cultivar × Injection treatment											
p-value	*0.4383*	*0.3146*	*0.4454*	*0.3342*	*0.5138*	*0.2473*	*0.2787*	*0.5051*	*0.9219*	*0.1494*	*0.3040*
Infection history × Rootstock cultivar × Injection treatment											
p-value	*0.8615*	*0.6966*	*0.1587*	*0.1629*	*0.7659*	*0.9152*	*0.0896*	*0.2461*	*0.2381*	*0.8032*	*0.3660*
Block											
p-value	*0.2260*	*0.0036*	*0.6738*	*0.0057*	*0.0091*	*0.1354*	*0.0221*	*0.0978*	*0.0042*	*0.0828*	*0.0261*

Different letters within columns indicate significant differences according to Tukey's honestly significant difference test. Letters are not shown when $P > 0.05$.

Supplementary Table 5. Macro and micronutrient content of leaves from 'Valencia' trees on different rootstocks with different infection histories and injection treatments in July 2024.

Factor	N (%)	P (%)	K (%)	Ca (%)	Mg (%)	S (%)	B (ppm)	Zn (ppm)	Mn (ppm)	Fe (ppm)	Cu (ppm)
Infection history											
Late-infected	2.69	0.18	1.36	4.06	0.37	0.27	85.6	26.4	37.5	69.9	10.9
Early-infected	2.73	0.18	1.39	4.09	0.38	0.27	89.0	25.0	37.4	72.3	11.8
p-value	*0.6521*	*0.8231*	*0.5806*	*0.9215*	*0.7013*	*1.0000*	*0.5708*	*0.5890*	*0.9663*	*0.7016*	*0.3677*
Rootstock cultivar											
US-812	2.60	0.18	1.34	4.02	0.39	0.27	87.5	27.8	37.9	74.4	10.9
US-942	2.82	0.19	1.41	4.13	0.36	0.26	87.1	23.6	37.0	67.8	11.8
p-value	*0.1895*	*0.5270*	*0.6763*	*0.6730*	*0.3157*	*0.4069*	*0.9487*	*0.4110*	*0.8930*	*0.6462*	*0.5509*
Injection treatment											
OTC-injected	2.67	0.18	1.36	3.96	0.37	0.27	85.3	27.0	38.6	72.4	11.4
Non-injected	2.74	0.18	1.39	4.19	0.38	0.27	89.4	24.4	36.3	69.8	11.3
p-value	*0.4885*	*0.8231*	*0.7203*	*0.2936*	*0.4100*	*0.4601*	*0.4120*	*0.3178*	*0.4343*	*0.6723*	*0.8939*
Infection history × Rootstock cultivar											
p-value	*0.5667*	*0.8231*	*0.9362*	*0.8331*	*0.7013*	*1.0000*	*0.7813*	*0.8040*	*0.8991*	*0.2583*	*0.6909*
Infection history × Injection treatment											
p-value	*0.3215*	*0.1532*	*0.2179*	*0.3821*	*0.3357*	*0.1778*	*0.3191*	*0.2423*	*0.7052*	*0.3219*	*0.3677*
Rootstock cultivar × Injection treatment											
p-value	*0.4180*	*0.2872*	*0.3032*	*0.6691*	*0.9385*	*0.4601*	*0.5584*	*0.3624*	*0.4343*	*0.7925*	*0.6909*
Infection history × Rootstock cultivar × Injection treatment											
p-value	*0.3795*	*0.8231*	*0.7802*	*0.3175*	*0.1770*	*1.0000*	*0.4385*	*0.2109*	*0.5873*	*0.0362*	*0.1768*
Block											
p-value	*0.7628*	*0.2052*	*0.1322*	*1.0000*	*0.8162*	*1.0000*	*1.0000*	*0.3529*	*0.2145*	*0.1935*	*0.5676*

Different letters within columns indicate significant differences according to Tukey's honestly significant difference test. Letters are not shown when P > 0.05.
